# Immune regulatory genes impact the hot/cold tumor microenvironment, affecting cancer treatment and patient outcomes

**DOI:** 10.3389/fimmu.2024.1382842

**Published:** 2025-01-22

**Authors:** Mengmeng Sang, Jia Ge, Juan Ge, Gu Tang, Qiwen Wang, Jiarun Wu, Liming Mao, Xiaoling Ding, Xiaorong Zhou

**Affiliations:** ^1^ Department of Immunology, School of Medicine, Nantong University, Nantong, China; ^2^ Department of Respiratory Medicine, Affiliated Nantong Hospital of Shanghai University, Nantong, China; ^3^ Department of Gastroenterology, Affiliated Hospital of Nantong University, Nantong, China; ^4^ Basic Medical Research Center, School of Medicine, Nantong University, Nantong, China

**Keywords:** tumor microenvironment, pan-cancer, hot/cold tumors, immunotherapy, pancreatic adenocarcinoma

## Abstract

**Background and aims:**

Immunologically hot tumors, characterized by an inflamed tumor microenvironment (TME), contrast significantly with immunologically cold tumors. The identification of these tumor immune subtypes holds clinical significance, as hot tumors may exhibit improved prognoses and heightened responsiveness to checkpoint blockade therapy. Nevertheless, as yet there is no consensus regarding the clinically relevant definition of hot/cold tumors, and the influence of immune genes on the formation of hot/cold tumors remains poorly understood.

**Methods:**

Data for 33 different types of cancer were obtained from The Cancer Genome Atlas database, and their immune composition was assessed using the CIBERSORT algorithm. Tumors were categorized as either hot or cold based on their distinct immune composition, ongoing immune response, and overall survival. A customized immunogram was created to identify important immunological characteristics. Kyoto Encyclopedia of Genes and Genomes and Hallmark pathway enrichment were evaluated through gene set variation analysis. Additionally, hub genes that regulate the tumor microenvironment were identified, and their expression patterns were analyzed using single-cell RNA sequencing. Furthermore, drug sensitivity and molecular docking analyses were performed to identify potential drug candidates capable of transforming cold tumors into hot tumors. For validation, a clinical cohort of patients diagnosed with pancreatic adenocarcinoma was examined using multiplex immunohistochemistry.

**Results:**

We were able to differentiate between hot and cold tumors in various types of cancer (bladder urothelial carcinoma, pancreatic adenocarcinoma, and cervical squamous cell carcinoma) by analyzing the presence of CD8+ T cells, activated natural killer cells, and M2-type macrophages, as well as the cytolytic activity and T cell proliferation. Hub genes that regulate the TME, including *PDCD1*, *CD276*, and *NT5E*, were discovered. The increased expression of *NT5E* and its prognostic significance were confirmed through multiplex immunohistochemistry in pancreatic adenocarcinoma. Finally, dasatinib and tozasertib were identified as drug candidates capable of converting cold pancreatic adenocarcinoma tumors into hot tumors.

**Conclusion:**

In this study, we developed a framework for discerning clinically significant immune subtypes across various cancer types, further identifying several potential targets for converting cold tumors into hot tumors to enhance anticancer treatment efficacy.

## 1 Introduction

Previous studies have classified tumors into immunologically hot and cold types based on the tumor microenvironment (TME), which comprises a mixture of malignant and stroma cells involved in a complex network of cellular and molecular interactions, among which immune cells are particularly important (1–3). It is thought that these characteristics impact the efficacy of immune checkpoint blockade (ICB) therapy. Cold tumors are defined by an immunosuppressive TME, characterized by a minimal immune infiltration, particularly CD8+ T cells and natural killer (NK) cells, leading to inadequate tumor control and poor response to immune checkpoint therapies. In contrast, hot tumors typically possess a TME with a prominent immune infiltration, and sometimes display a heightened response to ICB therapy (1–3).

Although significant advancements have recently been made in the definitions of cold/hot tumors and the understanding of their influence on cancer immunity, they remain incompletely understood (1–4). The main indicator of hot tumors is the presence of intratumoral CD8+ cytotoxic T lymphocytes (CTLs), which are major effector cells capable of recognizing and attacking tumor cells (5, 6). However, various immunosuppressive factors in the TME can cause dysfunction or exhaustion of CTLs and hinder their cytotoxic activity (5, 6). As such, although the infiltration of CTLs is a prerequisite, it is insufficient to determine whether the immune system can control tumor growth, or if a patient will respond favorably to ICB therapy. The application of biomarkers may help to predict the therapeutic efficacy of ICB. For example, patients with a high tumor mutational burden and tumor programmed cell death-ligand 1 (PD-L1) expression may respond better to immunotherapy targeting programmed cell death-1 (PD-1) than others. However, the predictive value of these biomarkers has been unsatisfactory in real-world practice (7–9). As such, it is crucial to develop an in-depth understanding of the immune composition of the TME and its impact on cancer immunity.

The priming and activation of CTLs can be facilitated by CD4+ T helper type 1 cells, NK cells, and certain subtypes of dendritic cells (6, 10–12). However, CTL-mediated antitumor immunity can be inhibited by immunosuppressive cells within the TME, such as M2 tumor-associated macrophages (TAMs), myeloid-derived suppressor cells (MDSCs), cancer-associated fibroblasts, and regulatory T cells (Tregs) (6, 10, 13, 14). Other studies have further demonstrated that TAMs predominantly have an M2-type phenotype and promote cancer progression by producing angiogenic and anti-inflammatory factors (15). In contrast, it is thought that M1-type TAMs possess anti-tumorigenic activities by secreting pro-inflammatory mediators or modulating the anticancer activity of other immune cells. For example, the release of extracellular vesicles by M1 macrophages facilitates the repolarization of M2 to M1 macrophages, thereby improving the effectiveness of anti-PD-L1 treatment in mice (16). It has further been recognized that the TME is constantly influenced by various tumor cell-intrinsic and -extrinsic factors including tumor cell immunogenicity, tumor-infiltrating T cell repertoire, T cell proliferation, and T cell functional exhaustion, all of which impact the establishment of the TME and influence its antitumor immune response (17). As such, a classification framework that incorporates immune composition and critical immunological traits is needed for determining hot/cold immune phenotypes with clinical relevance.

Transforming cold tumors into hot tumors is considered a promising strategy to improve treatment outcomes of immunotherapy and possibly conventional therapy (17). Several recent studies have suggested means to help achieve this goal. For example, *KRAS* mutations in pancreatic cancer trigger the constitutive activation of downstream signaling (18). This activation increases PD-L1 expression and recruits various immunosuppressive cells, thereby inhibiting anticancer T cell responses (19). These effects lead to a cold tumor milieu in *KRAS*-mutant pancreatic adenocarcinoma (PAAD) (20). As such, the conversion of cold tumors into hot tumors in *KRAS*-mutant PAAD could be induced by treatment with *KRAS* inhibitors (21). Furthermore, augmented autophagy in pancreatic cancer cells results in the degradation of major histocompatibility complex (MHC) class I molecules, which reduces the presentation of neoantigens by tumor cells. As a result, inhibition of autophagy has been found to suppress tumor growth by enhancing MHC class I expression and CTL-mediated tumor cell killing (22). These results suggest that targeting the immunosuppressive TME can convert cold tumors into hot tumors, thereby promoting anticancer immune responses.

The present study outlines a method for tumor immune phenotyping by delineating overall immune cell components and critical immunological features in the TME. We utilized data from The Cancer Genome Atlas (TCGA) database, which contains data on 33 different types of cancers, to evaluate immune cell compositions using the CIBERSORT algorithm. Furthermore, we distinguished between hot or cold tumors by examining the proportions of intratumoral CD8+ T cells, activated NK cells, and M2 macrophages, as well as the scores of cytolytic activity and T cell proliferation. Potential biomarkers were also identified and validated, which could help to distinguish tumor immune phenotypes and may have potential as targets to promote the transition of tumors from cold-to-hot states.

## 2 Materials and methods

### 2.1 Data sources and preprocessing

The transcripts per million expression data of 33 cancer types were obtained from the UCSC database and normalized. Only data of cancer samples were used in this investigation, and all normal data were excluded. Clinical metadata, mutation annotation data, and copy number variation data were also obtained from the UCSC database. Single-cell RNA sequencing (scRNA-seq) datasets of PAAD, including CRA001160 (23), GSE111672 (24), GSE141017 (25), GSE148673 (26), GSE154778 (27), GSE158356 (28), GSE162708 (29), and GSE165399 (30), were downloaded from the Gene Expression Omnibus (GEO: https://www.ncbi.nlm.nih.gov/geo/) database and to identify the gene expression in various cell types. Immunohistochemistry (IHC) data were further downloaded from the Human Protein Atlas (HPA, http://www.proteinatlas.org) database to verify protein expression in PAAD.

### 2.2 Immune infiltration analysis and clustering

The CIBERSORT algorithm (31) of the IOBR packages (v.0.99.9) (32) was employed to measure the infiltration of 22 immune cell types. Using the R package “ConsensusClusterPlus” (v 1.64.0) (33), we identified various clusters that differ in terms of immune infiltration by consensus clustering for each cancer type. The number of clusters was determined by the k value and the area under the cumulative distribution function curve. To ensure the accuracy of our classification results, we repeated this step 1,000 times. The Single-sample Gene Set Enrichment Analysis (ssGSEA), as implemented in the R package GSVA (version 0.99.9) (34), was utilized to quantify 13 immune function scores derived from the work of He et al. (35), including T cell proliferation (36) and MDSCs (37). Based on the infiltration levels of CD8+ T cells, activated NK cells, and M2-type macrophages, as well as the scores for cytolytic activity and T cell proliferation, we categorized the clusters into two distinct groups: “hot-immune” and “cold-immune.” Additionally, we utilized several computational algorithms, including the Tumor Immune Estimation Resource (TIMER) (38), EPIC (39), Microenvironment Cell Populations-counter (MCP-counter) (40), xCELL (41), and quanTIseq (42), to quantify immune cell infiltration and to identify key immune cell types associated with hot and cold tumor phenotypes.

### 2.3 Cox proportional hazards regression model

Based on the identified cold and hot tumor types, we performed a univariate Cox regression model in the *survival* (v 3.2-7) package (43) to analyze the prognostic relationship between cold and hot tumors. Tumors with p-values <0.05 were retained for further analysis.

### 2.4 Immune regulatory and checkpoint gene analysis

We sourced immune regulatory genes, including chemokines, receptors, major histocompatibility complex (MHC) genes, immunoinhibitors, and immunostimulators, from the Sangerbox database (44). Additionally, we obtained immune checkpoint genes, encompassing both inhibitory and stimulatory genes, based on the study by Thorsson et al. (45). We conducted an analysis of the correlation between immune regulatory and checkpoint genes and the infiltration of 22 immune cell types, as estimated by the CIBERSORT algorithm, across various cancer types.

### 2.5 Gene set variation analysis

Gene expression enrichment was evaluated through GSVA analysis under both unsupervised and parameter-free conditions (34). GSVA was employed to examine the various Kyoto Encyclopedia of Genes and Genomes (KEGG) and hallmark pathways between hot and cold tumor types. Downloads for KEGG and hallmark gene sets were obtained from the Molecular Signatures Database (https://www.gsea-msigdb.org/gsea/msigdb/index.jsp), and hypergeometric p-values were adjusted using Benjamini–Hochberg multiple testing correction.

### 2.6 Gene set enrichment analysis

The GSEA algorithm was applied to identify expression profiles that could either activate or suppress hallmark pathways between high- and low-survival groups. After 100 permutations, an enriched gene set was obtained based on a p-value <0.05 and a false discovery rate of 0.25.

### 2.7 Analysis of drug sensitivity and responsiveness to immunotherapy in PAAD

Data on drug sensitivity were obtained from The Genomics of Drug Sensitivity in Cancer database (https://www.cancerrxgene.org/). The half maximal inhibitory concentration (IC_50_) values of each drug were downloaded using the R package “oncoPredict” (v 0.2) (46). Subsequently, we conducted a correlation analysis between drug sensitivity and the genes that control immune responses. Furthermore, we computed the dissimilarities in drug sensitivity among patients with hot and cold tumors. This investigation of the correlation between drug sensitivity and immune regulatory gene expression in various patient groups and subtypes was conducted with the aim of identifying potential treatments to provide new insights into personalized therapeutic strategies for patients with PAAD. The tumor immune dysfunction and exclusion (TIDE) algorithm was used to model the tumor immune evasion of hot/cold-immune tumors (47). The processed RNA expression levels of five patients with cancer were uploaded to the online TIDE database website (http://tide.dfci.harvard.edu/) to derive the TIDE score of each patient for predicting immunotherapy response.

### 2.8 Immunological feature analysis

The “ESTIMATE” R package (v 1.0.13) (48) was further applied to calculate the three immune-related scores, namely StromaScore, ImmuneScore, and ESTIMATEScore, for each patient. Additionally, we conducted an analysis of the anticancer immune response using the Tracking Tumor Immunophenotype (TIP) database (http://biocc.hrbmu.edu.cn/TIP/) (49).

### 2.9 Docking drugs and protein molecules

The protein structures corresponding to several identified genes were downloaded from the Protein Data Bank database (https://www.rcsb.org/) (50) and pretreated with the UCSF Chimera (v 1.15). This included adding hydrogen, assigning partial charges and protonation states, and energy minimization (51). The chemical structure of active drug compounds was downloaded from the ZINC15 database (https://zinc15.docking.org/) (52). All compounds were subsequently docked into the binding sites of target proteins using the software DOCK (v 6.10), and visualized using the UCSF Chimera (v 1.14) and LigPlus (v 2019).

### 2.10 Multiplex immunohistochemistry

Paraffin-embedded sections of PAAD were obtained from the Affiliated Hospital of Nantong University (Nantong, China). This study was approved by the Ethical Committee of the Affiliated Hospital of Nantong University. Immunofluorescent analysis was performed to identify and assess the protein expression of pancytokeratin (PANCK; tumor epithelium marker), CD8, 5’-nucleotidase ecto (NT5E; CD73), and CD163 (M2 macrophage marker) in tumor tissues. All antibodies were purchased from AiFang Biological, Changsha City, China (product numbers AF20164, AF20211, AF301239, and AF20010 for PANCK, CD8, NT5E, and CD163, respectively). Paraffin-embedded sections were treated with xylene, briefly washed in a graded series of ethanol (100%, 95%, 85%, 80%, 75% ethanol), and subsequently washed with distilled water. The tissue slices were treated with antigen retrieval buffer with ethylenediaminetetraacetic acid (pH 9.0), and heated in a microwave oven. Next, the slices were immersed in a 3% hydrogen peroxide solution and incubated at room temperature for 15 min to eliminate the endogenous peroxidase. Subsequently, they were washed with phosphate-buffered saline (pH 7.4) in a decolorization shaker for 5 min, and treated with goat serum for blocking at room temperature for 30 min. Thereafter, staining was performed according to the instructions provided by the manufacturer (3-Color Multiple fluorescence Kit; AiFang Biological). Images were captured using the ECLIPSE Ci series microscope (Nikon, Tokyo, Japan), and were analyzed with the HALO image analysis platform (Indica labs, Albuquerque, NM).

### 2.11 Statistical analysis

R 4.0.5 software (R Project for Statistical Computing, Vienna, Austria) was used for data processing, statistical analysis, and plotting. The correlation between two continuous variables was evaluated using Pearson’s correlation coefficients, while the chi-squared test was used to compare categorical variables, and the Wilcoxon rank-sum test or *t*-test was used to compare continuous variables.

## 3 Results

### 3.1 Pan-cancer clustering of tumor immune subtypes with clinical relevance

**Figure 1:**
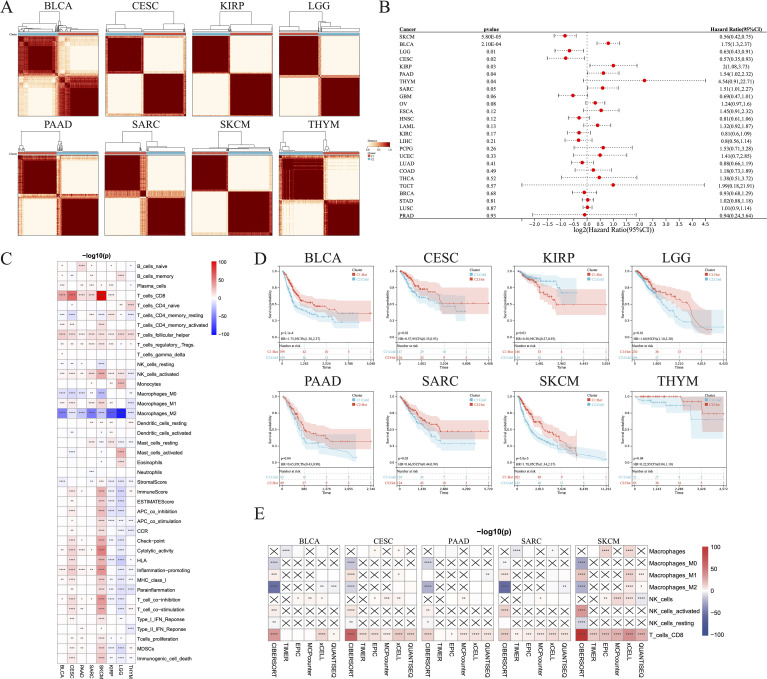
Identification of hot and cold tumors based on immune composition and activity. **(A)** Patients were divided into clusters based on immune composition assessed using the CIBERSORT algorithms in pan-cancer. **(B)** Forest map showing pan-cancer prognostic differences among the different clusters. **(C)** Infiltration of various immune cells and immunological feature scores in hot and cold tumors of the indicated cancer types. **(D)** KM survival analysis of hot and cold tumors. **(E)** The TIMER, EPIC, MCP-counter, xCell, and quanTIseq algorithms were used to estimate the immune composition in hot versus cold tumors of the indicated cancer types (*p < 0.05, **p < 0.01, ***p < 0.001, ****p < 0.0001). CI, confidence interval; ns, not significant.

The CIBERSORT algorithm was used to assess the infiltration levels of 22 immune cell types and subsequently perform a consensus cluster analysis. The 33 types of cancer samples were grouped into 2–9 clusters based on the type of cancer. Cumulative distribution function curves of the consensus score matrix and proportion of ambiguous clustering statistics were used to determine the optimal number for the 33 cancer types (**Figure 1A**). Using the Cox model, we estimated the survival probability and determined the significance of clustering in each cancer type. Kaplan–Meier (KM) survival analysis revealed statistically significant findings for only eight of the 33 cancer types: skin cutaneous melanoma (SKCM), bladder urothelial carcinoma (BLCA), brain lower grade glioma (LGG), cervical squamous cell carcinoma and endocervical adenocarcinoma (CESC), kidney renal papillary cell carcinoma (KIRP), PAAD, thymoma (THYM), and sarcoma (SARC) (**Figure 1B**).

We were able to differentiate the aforementioned clusters into hot and cold tumor types by considering the infiltration of CD8+ T cells, activated NK cells, and M2 macrophages, along with the scores of cytolytic activity and T cell proliferation. Increased numbers of CD8+ T and activated NK cells, higher scores of cytolytic activity and T cell proliferation, and fewer M2 macrophages were observed in hot tumors compared with cold tumors in BLCA (**Figure 1C**; **Supplementary Figure 1A**), CESC (**Figure 1C**; **Supplementary Figure 1B**), PAAD (**Figure 1C**, **Supplementary Figure 1C**), SARC (**Figure 1C**, **Supplementary Figure 1D**), and SKCM (**Figure 1C**, **Supplementary Figure 1E**). These five types of cancer all exhibited a strong consistency in the cold versus hot immune states, whereas the remaining three types had only a few distinct features; for example, hot LGG tumors exhibited lower cytolytic activity and T cell proliferation than cold LGG tumors (**Figure 1C**; **Supplementary Figures 1F–H**). In addition, there was no significant disparity in the abundance of CD8+ T cells between hot and cold THYM tumors, while several immune-related scores were inversely correlated with the presence of hot THYM tumors. KM analysis indicated that hot tumors of seven of the eight cancer types were associated with better survival than cold tumors, whereas the opposite was true for KIRP (**Figure 1D**). These observations led to the selection of BLCA, CESC, PAAD, SARC, and SKCM for further investigation, due to their manifestation of a comparable hot/cold immune state, with a more favorable prognosis observed for hot tumors. To verify the stability and robustness of the results obtained using the CIBERSORT algorithm, we used five additional algorithms, including TIMER, EPIC, MCP-counter, xCell, and quanTIseq, to ensure that the two consensus clusters were not biased by the analytical algorithm (**Figure 1E**).

**Figure 2:**
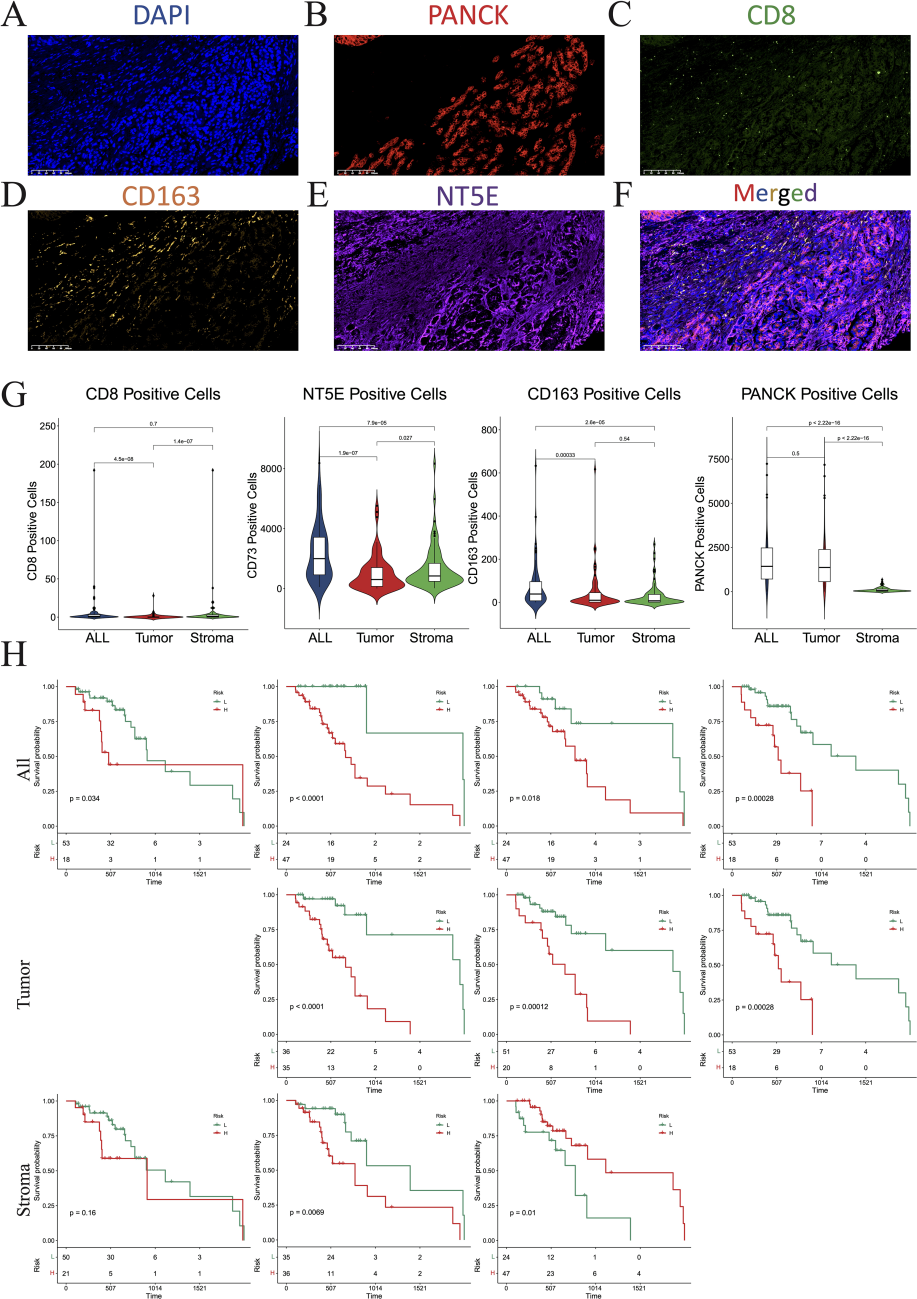
Multiplex immunohistochemistry (mIHC). **(A–F)** Numbers of cells expressing DAPI **(A)**, PANCK **(B)**, CD8 **(C)**, CD163 **(D)**, NT5E **(E)**, and merged **(F)**. **(G)** Violin plots showing the differences in the numbers of CD8, NT5E, CD163, and PANCK-positive cells between the whole section, tumoral region, and stromal region. **(H)** KM plot showing that the number of CD8-, NT5E-, CD163-, and PANCK-positive cells affected prognosis. DAPI, 4’,6-diamidino-2-phenylindole; PANCK, pancytokeratin.

To further verify the distinct immune compositions in hot and cold tumors and their implications on patient survival, we conducted an mIHC analysis using a tissue array of 71 patients with PAAD to identify the expression levels of CD8 (a marker of CD8 T cells), CD163 (a marker of M2 macrophages), and PANCK (a marker of cancer cells) (**Figures 2A–D**). This study revealed that CD8+ cells were sparsely distributed in the stromal regions, and were scarcely detected in some PAAD samples. Additionally, the total number of CD8+ T cells was found to be inversely correlated with overall survival (**Figure 2E**), indicating a compromised CTL response in PAAD. CD163+ cells were identified in both the tumor and stroma regions, as depicted in **Figure 2F**. Remarkably, an increased presence of CD163+ cells in tumor regions was correlated with reduced survival rates, whereas a greater abundance of stromal CD163+ cells correlated with improved patient survival, as shown in **Figure 2G**. These findings suggest that the CD163+ M2 macrophages within tumor regions are primarily responsible for protumoral functions. As expected, PANCK+ cells were exclusively identified in the tumoral region (**Figure 2H**). Interestingly, a decrease in PANCK+ cell numbers was found to be correlated with enhanced survival rates (**Figure 2H**), indicating that a lower tumor purity or a higher stroma component within the TME may be linked to the presence of hot tumors in PAAD. According to these findings, we suggest that the levels of M2 macrophage infiltration and tumor purity, which can be simply assessed through routine immunohistochemistry (IHC) in clinical settings, may serve as biomarkers for differentiating hot and cold tumors and predicting the prognosis of patients with PAAD.

### 3.2 Analysis of immune subtype-associated somatic mutations

**Figure 3:**
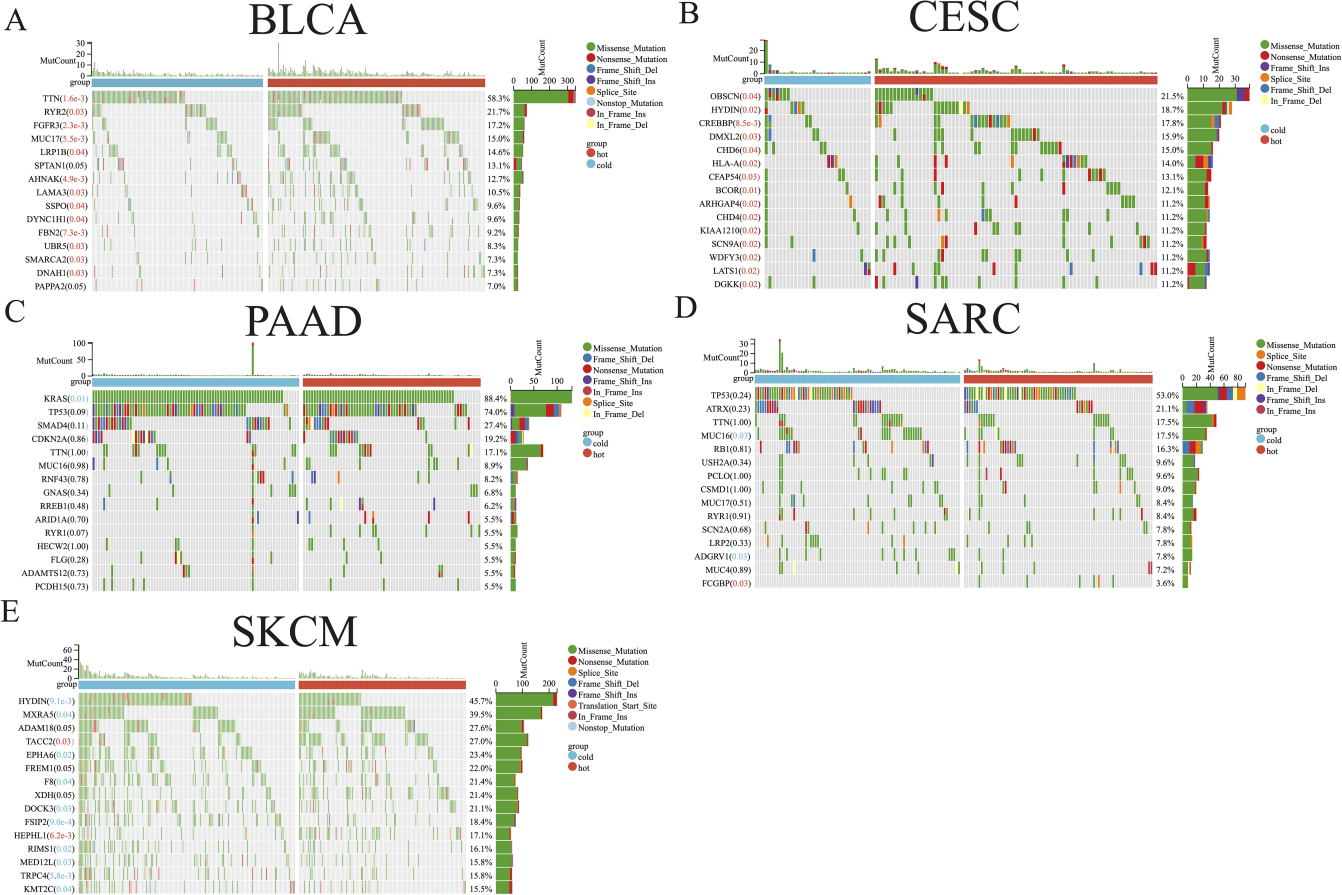
Somatic mutation analysis of hot and cold tumors. Waterfall plot showing differences in somatic mutation frequency between hot and cold tumors in BLCA **(A)**, CESC **(B)**, PAAD **(C)**, SARC **(D)**, and SKCM **(E)**. Significantly higher mutation frequencies in hot or cold tumors are highlighted in red and blue text, respectively.

The distribution of somatic mutations in the five aforementioned cancer types was further examined, allowing comparisons of the mutation frequencies in hot and cold tumors (**Figure 3**). Only the top 15 genes in each cancer type were shown due to their high mutation frequency. Genes with a mutation frequency exceeding 20% were as follows: *TTN* (58.3%) and *RYR2* (21.7%) in BLCA, *OBSCN* (21.5%) in CESC, *KRAS* (88.4%), *TP53* (74.0%), *SMAD4* (27.4%) in PAAD, *TP53* (53.0%) and *ATRX* (21.2%) in SARC, *HYDIN* (45.7%), *MXRA5* (39.5%), *ADAM18* (27.6%), *TACC2* (27.0%), *EPHA6* (23.4%), *FREM1* (22.0%), *F8* (21.4%), *XDH* (21.4%), and *DOCK3* (21.1%) in SKCM. We identified a significant difference between hot and cold tumors in the mutation frequency of all top 15 genes in BLCA and CESC, with hot tumors containing more mutations (**Figures 3A, B**). In PAAD, *KRAS* was the only gene more frequently mutated in cold tumors than in hot tumors (**Figure 3C**). In SARC, hot tumors had more mutations in *FCGBP*, but fewer mutations in *MUC16* and *ADGRV1*, than cold tumors (**Figure 3D**). In SKCM, *TACC2* and *HEPHL1* were more frequently mutated in hot tumors, whereas most other genes were more frequently mutated in cold tumors (**Figure 3E**).

### 3.3 Analysis of the immune landscape and responsiveness to immunotherapy

**Figure 4:**
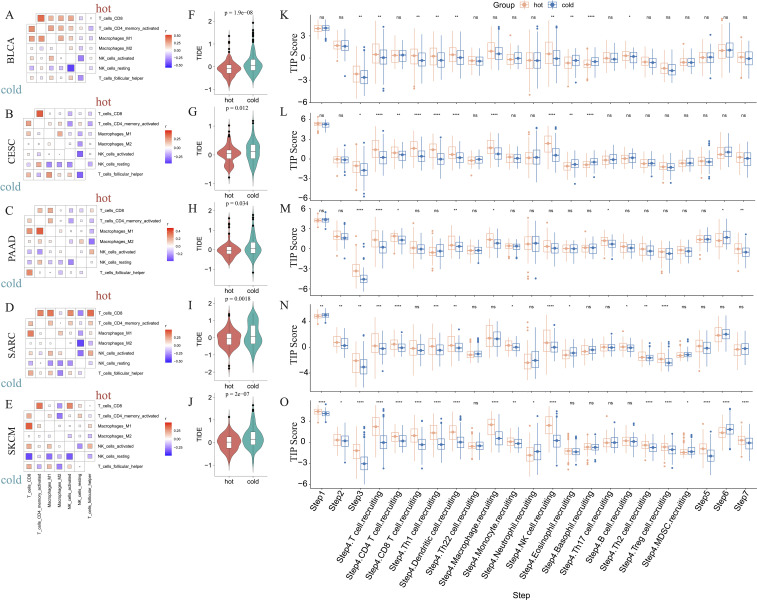
Correlations between immune cells and immunological feature scores. Correlations between six immune cell infiltrations in BLCA **(A)**, CESC **(B)**, PAAD **(C)**, SARC **(D)**, and SKCM **(E)**. Comparison of the TIDE scores of hot and cold tumors of BLCA **(F)**, CESC **(G)**, PAAD **(H)**, SARC **(I)**, and SKCM **(J)** (ns: p > 0.05, *p < 0.05, **p < 0.01, ***p < 0.001, ****p < 0.0001). The difference in TIP immune activity scores between hot and cold tumors in BLCA **(K)**, CESC **(L)**, PAAD **(M)**, SARC **(N)**, and SKCM **(O)** (ns, p > 0.05, *p < 0.05, **p < 0.01, ***p < 0.001, ****p < 0.0001).

We further determined the correlations between diverse immune cells in the five abovementioned cancer types, identifying a different pattern between hot and cold tumors for each cancer type (**Figures 4A–E**; **Supplementary Figures 2A–E**). For example, CD8+ T cells in hot BLCA tumors were positively correlated with activated NK cells, whereas this correlation was insignificant in cold BLCA tumors (**Figure 4A**). Follicular helper T cells universally exhibited a positive correlation with CD8+ T cells, particularly in cold tumors (**Figures 4A–E**). The TIDE score has been widely used to predict resistance to immunotherapy, with higher TIDE scores indicating a higher potential for immune escape and lower immunotherapy response rates (47). We further calculated the TIDE score to predict responsiveness to immunotherapy, finding that hot tumors were more likely to respond to immunotherapy than cold tumors in all five of the examined cancer types (**Figures 4F–J**). We further examined the crosstalk of certain critical stimulatory and inhibitory components of cancer immunity in hot and cold tumors. These findings suggested that CD8+ CTLs were positively correlated with most of the immune suppressive signatures, such as MDSC presence, T cell co-inhibition, antigen-presenting cell co-inhibition, and inflammation-promotion (**Supplementary Figures 2F–J**), suggesting that these factors may collectively protect tumor cells from attack by CTLs.

Furthermore, we evaluated the activities of multiple steps in anticancer immune response using the TIP database. These findings suggested that hot tumors generally displayed higher activity scores than cold tumors, particularly in SARC and SKCM (**Figures 4K–O**). Further, the priming stages of anticancer immune response were significantly inhibited in cold tumors. For example, hot tumors from multiple cancer types exhibited higher activity scores than cold tumors for step 1 (release of cancer cell antigens), step 2 (cancer antigen presentation), and step 3 (priming and activation). Further, in step 4 (trafficking of immune cells to tumors), we consistently found that hot tumors exhibited higher scores for various immune cell types, including CD8+ T cells and NK cells. In step 5 (infiltration of immune cells into tumors), only cold SKCM tumors exhibited lower activity scores than hot tumors, whereas in the other four types of cancers, cold and hot tumors displayed similar scores, indicating that most cold tumors were not inherently immune-excluded relative to hot tumors. The scores for step 7 (activity of killing cancer cells) were higher in PAAD and SKCM hot tumors, and were comparable between BLCA, CESC, and SARC hot and cold tumors. Interestingly, the scores for step 6 (recognition of cancer cells by T cells) were consistently lower in hot tumors than cold tumors, suggesting that insufficient neoantigen recognition by T cell receptors may be the critical immunosuppression factor in hot tumors despite the increased T cell recruitment (**Figures 4K–O**).

### 3.4 Establishing an immunogram for hot and cold tumors

**Figure 5:**
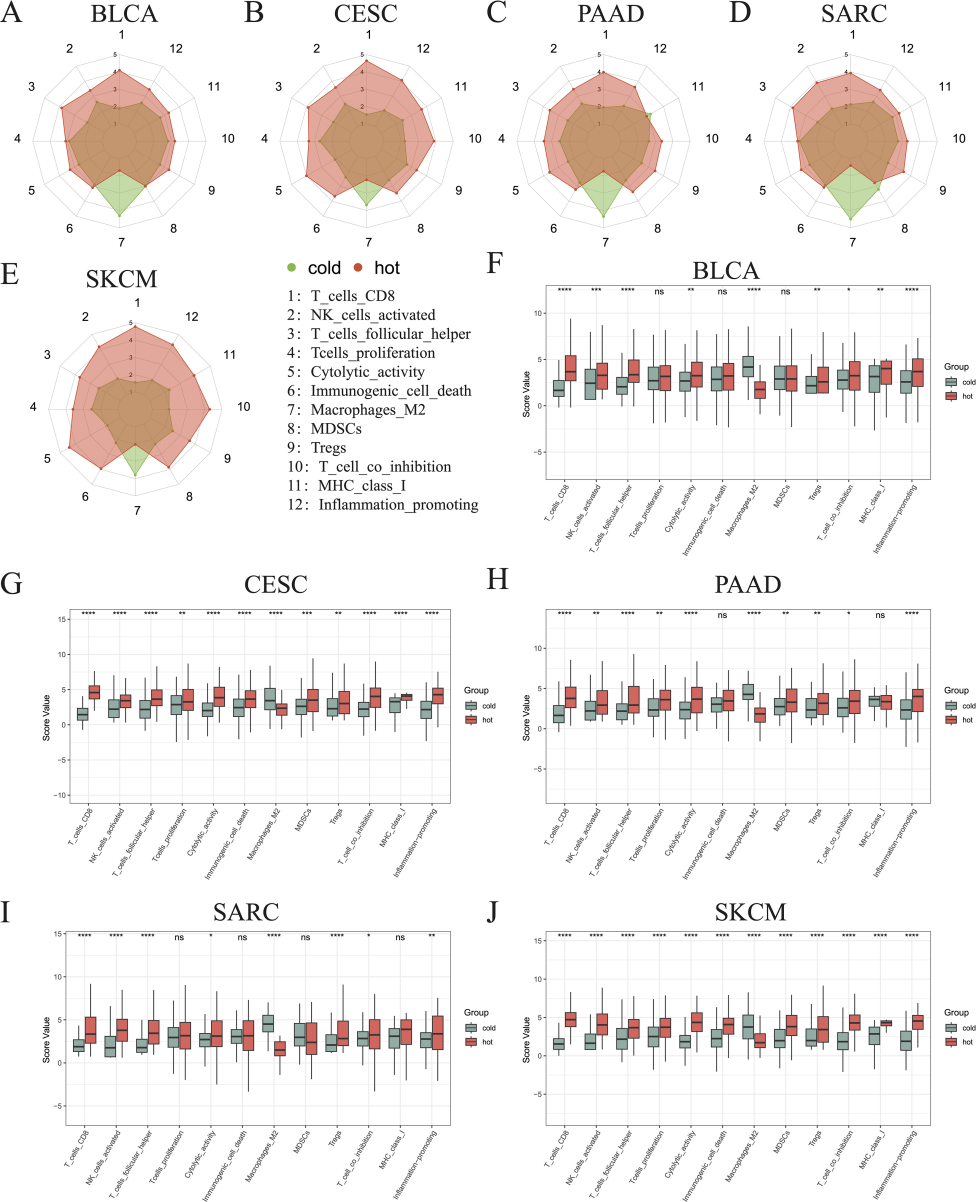
Immunograms showing the primary characteristics of cold and hot tumors. Radar charts showing the 12 main characteristics of cold and hot tumors of BLCA **(A)**, CESC **(B)**, PAAD **(C)**, SARC **(D)**, and SKCM **(E)**. Histogram showing the 12 main characteristics of cold and hot tumors of BLCA **(F)**, CESC **(G)**, PAAD **(H)**, SARC **(I)**, and SKCM **(J)** (ns, p > 0.05, *p < 0.05, **p < 0.01, ***p < 0.001, ****p < 0.0001).

To better describe the immune landscape of each tumor type, we developed an immunogram by incorporating seven antitumoral immune parameters (CD8+ T cells, activated NK cells, follicular helper T cells, T cells proliferation, cytolytic activity, immunogenic cell death, MHC class I) and five protumoral immune parameters (M2 macrophages, MDSCs, Tregs, T cell co-inhibition, inflammation-promotion). Consequently, a 12-axis radar plot was generated to visualize the immune state of hot and cold tumors (**Figures 5A–E**). We further found that only the values of axis 7 (M2 macrophages) were higher in all cold tumors (**Figures 5A–E**), while those of axis 8 (MDSCs) were higher in cold tumors of the BLCA and SARC groups (**Figures 5A, D**). Surprisingly, the values of axis 11 (MHC class I) were higher in cold tumors of PAAD (**Figure 5C**), although the difference was not statistically significant (**Figures 5F–J**). Concerning other parameters, higher values were consistently observed in hot tumors, regardless of cancer type. Collectively, these results indicate a similar pattern in all five cancer types, i.e., greater numbers of CD8+ T cells and follicular helper T cells and fewer M2 macrophages in hot tumors compared with cold tumors (p < 0.0001) (**Figures 5F–J**). Of note, the pattern was not shared by the other three types of cancer, namely KIRP, LGG, and THYM (**Supplementary Figures 3A–C**).

### 3.5 Differentially expressed genes and GSVA analysis

**Figure 6:**
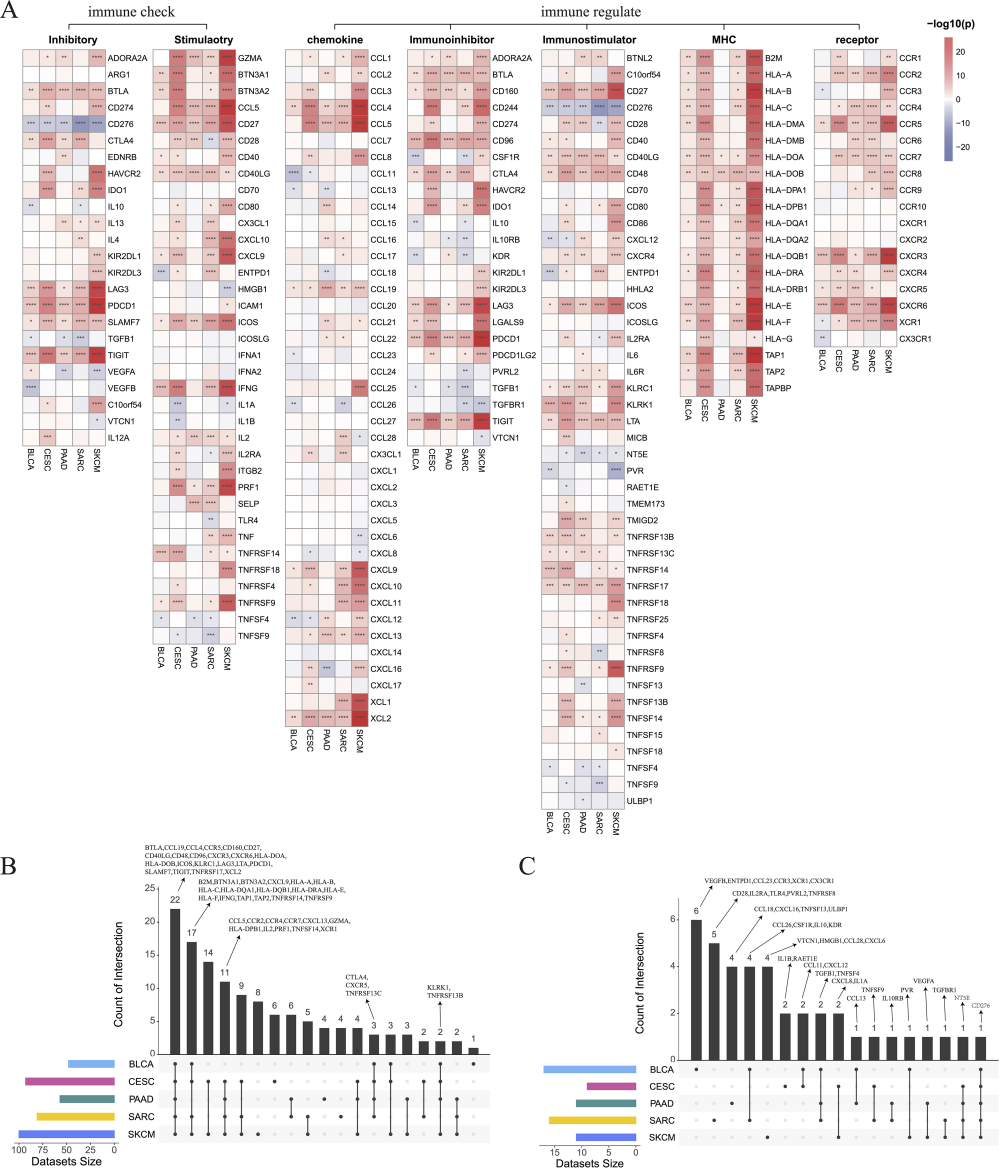
Differentially expressed immune genes between hot and cold tumors. **(A)** Heatmap showing the differentially expressed immune checkpoint and immune regulatory genes between hot and cold tumors of BLCA, CESC, PAAD, SARC, and SKCM. **(B)** Upset plot showing the upregulated immune genes in hot tumors of BLCA, CESC, PAAD, SARC, and SKCM. **(C)** Upset plot showing the downregulated immune genes in hot tumors (*p < 0.05, **p < 0.01, ***p < 0.001, ****p < 0.0001).

We selected 150 immune regulator genes and 60 immune checkpoint genes based on the results of prior studies conducted by Shen et al. (44) and Thorsson et al. (45), and compared their expression between our hot and cold tumor datasets (**Figure 6A**). The findings revealed that 22 genes, including key immune checkpoint molecules such as *PDCD1*, *TIGIT*, and *LAG-3*, were significantly upregulated in hot tumors across all five cancer types (**Figure 6B**). Additionally, the expression of *CD276* was found to be elevated in cold tumors across all five cancer types, while *NT5E* exhibited higher expression levels in cold tumors of the CESC, PAAD, SARC, and SKCM groups (**Figure 6C**). Other DEGs between hot and cold tumors are presented in **Figure 6C**.

**Figure 7:**
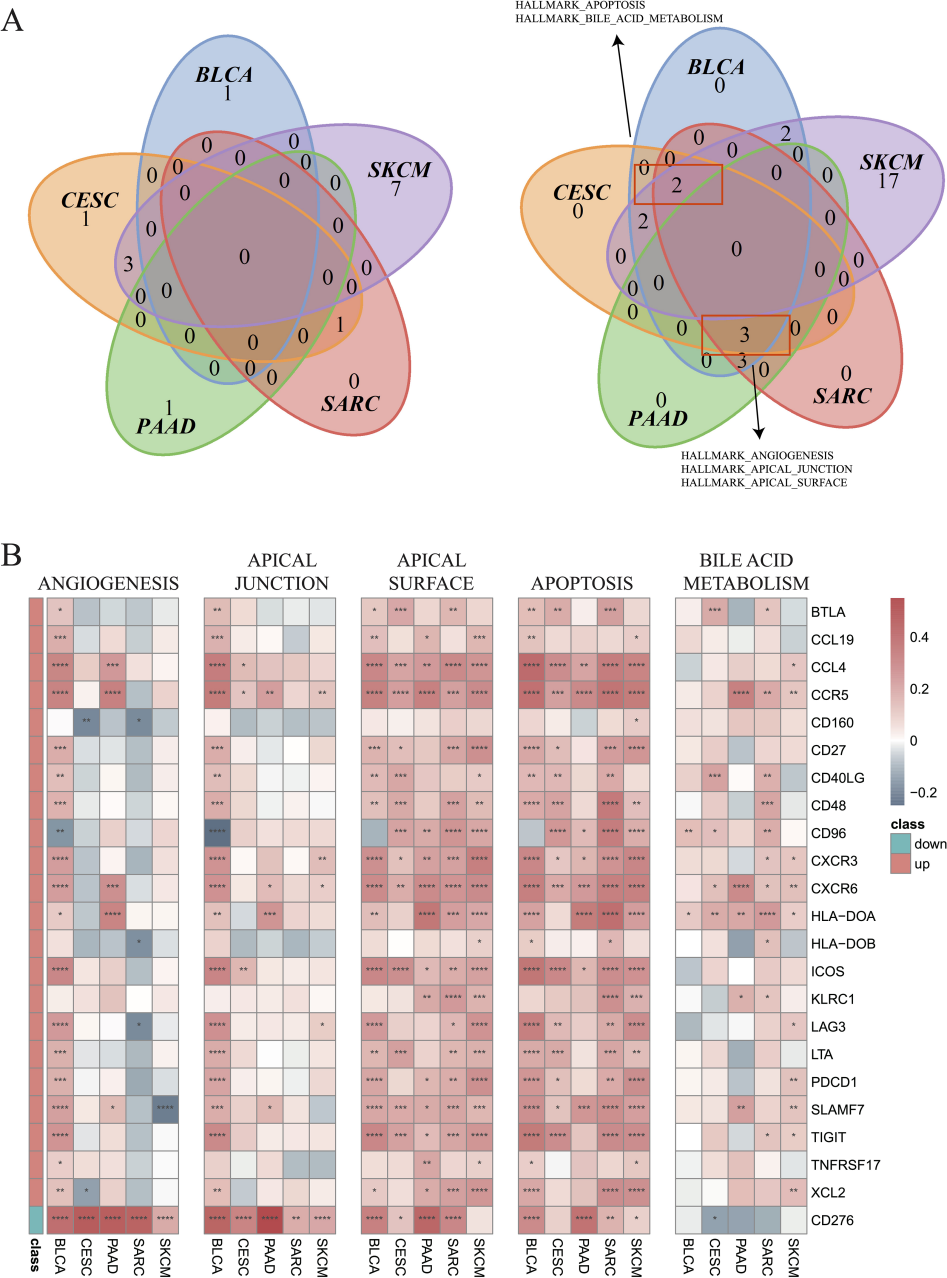
Enrichment of hallmark pathways between hot and cold tumors. **(A)** Venn plots showing the different hallmark pathways between hot and cold tumors of the indicated cancer types (left: upregulated in hot tumors, right: downregulated in hot tumors). **(B)** Correlations between 23 genes and five pathways in the indicated cancer types (*p < 0.05, **p < 0.01, ***p < 0.001, ****p < 0.0001).

Next, GSVA was employed to investigate the differential enrichment of hallmark pathways in hot and cold tumors (**Supplementary Figures 4A–E**). Our analysis revealed that the apoptosis and bile acid metabolism pathways were enriched in cold tumors of BLCA, CESC, SARC, and SKCM (**Figure 7A**); while the angiogenesis, apical junction, and apical surface pathways were enriched in cold tumors of BLCA, CESC, PAAD, and SARC (**Figure 7A**). Correlation analysis was further performed using the 23 shared DEGs (22 upregulated plus one downregulated gene in hot tumors compared with cold tumors from all five cancer types), in addition to the GSVA scores of the five enriched pathways. Interestingly, almost all DEGs were positively correlated with the apical surface and apoptosis pathways in all cancer types, indicating that these genes were actively involved in the regulation of these two pathways. In BLCA, numerous DEGs, except for *CD160* and *CD96*, were positively correlated with the angiogenesis and apical junction pathways; however, this correlation was not observed in the other four types of cancer (**Figure 7B**). We further performed GSVA on the KEGG pathways in hot and cold tumors separately, the results of which are shown in **Supplementary Figures 5A–H**.

### 3.6 Correlation analysis between immune genes and cells

**Figure 8:**
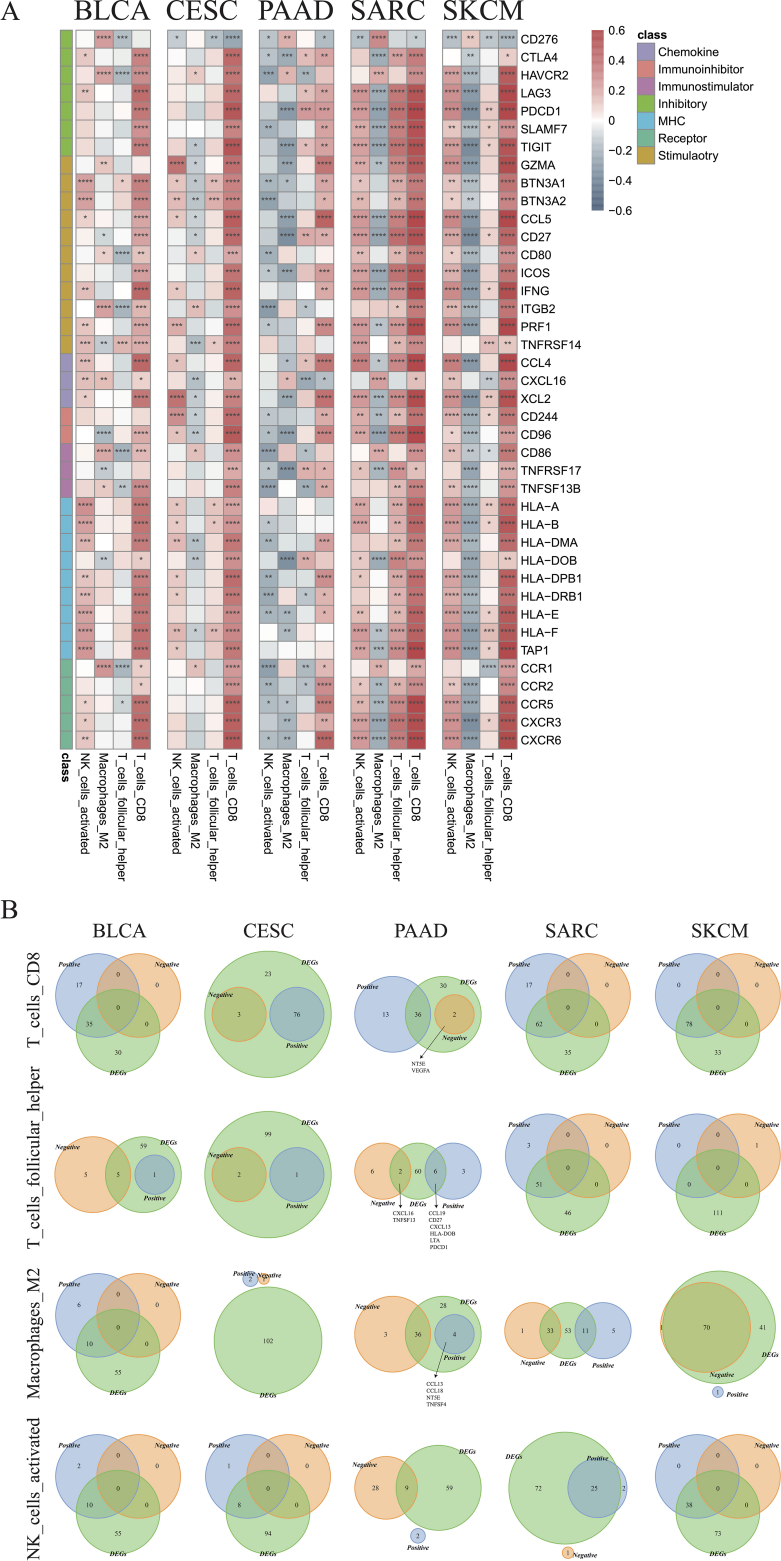
Identification of critical immune regulators. **(A)** Correlations between the expression of immune genes and infiltration of CD8+ T cells, follicular helper T cells, M2 macrophages, and activated NK cells in the indicated cancer types. **(B)** Venn plots showing the intersections of differentially expressed genes and immune cell-associated genes in CD8+ T cells, follicular helper T cells, M2 type macrophages, and activated NK cells in the indicated cancer types (*p < 0.05, **p < 0.01, ***p < 0.001, ****p < 0.0001).

Correlations between immune cell infiltration and the levels of previously indicated immune regulatory genes (44, 45) were further examined (**Supplementary Figure 6**). The genes with strong correlations to the indicated immune cell types are illustrated in **Figure 8A**. Most selected genes were positively correlated with CD8+ T cells in all cancer types, except for *CD276*, which was negatively correlated with CD8+ T cells. *CD276* was also negatively correlated with activated NK cells, but positively correlated with M2 macrophages in most cancer types. Positive correlations between most genes and activated NK cells were observed in BLCA, CESC, SARC, and SKCM. Nevertheless, an opposite trend was observed in PAAD, indicating an impaired recruitment of activated NK cells under these conditions. M2 macrophages were also found to be negatively associated with most genes in SKCM, PAAD, SARC, and CSEC (**Figure 8A**). The Venn plots in **Figure 8B** demonstrate the distribution of DEGs positively or negatively associated immune cells, specifically CD8+ T cells, M2 macrophages, activated NK cells, and follicular helper T cells. In PAAD, we further found that *NT5E* and *VEGFA* may negatively regulate CD8+ T cells, while *CCL13*, *CCL18*, *NT5E*, and *TNFSF4* might positively regulate M2 macrophages (R > 0.2). Other genes that may regulate other immune cells are listed in **Supplementary Table 1**.

### 3.7 Expression of *NT5E* and *CD276* and their impact on prognosis of PAAD

**Figure 9:**
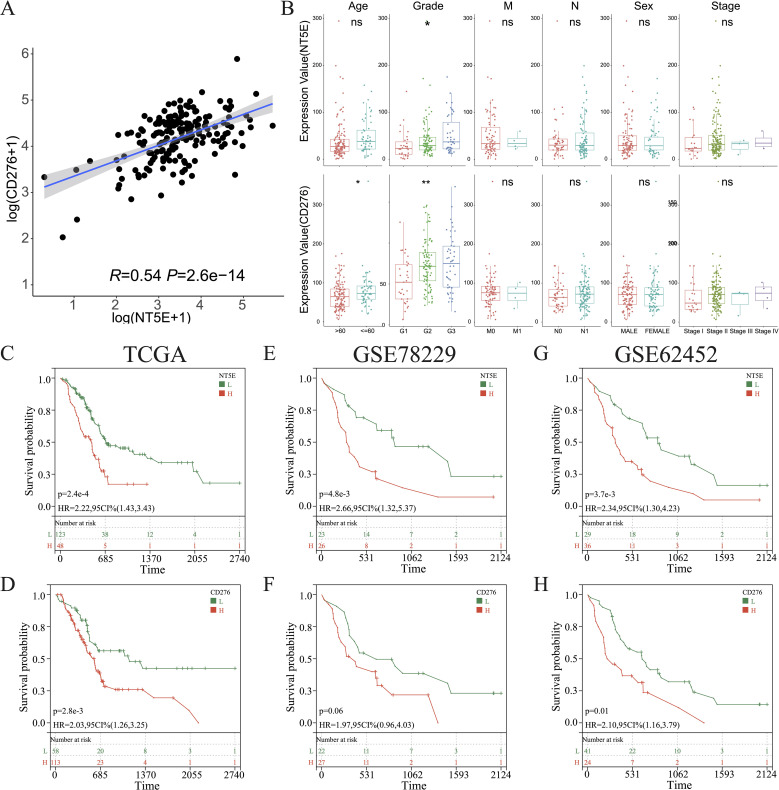
Clinical characterization of *CD276* and *NT5E* in PAAD. **(A)** Correlations between the levels of *CD276* and *NT5E* in PAAD. **(B)** Comparison of the expression of *CD276* and *NT5E* between patients with PAAD grouped by clinicopathological characteristics. **(C, E, G)** KM survival curve of OS between patients with high and low *NT5E* expression in TCGA databases GSE78229, and GSE62452. **(D, F, H)** KM survival curve of OS between patients with high and low *CD276* expression in the three aforementioned datasets. (ns, p > 0.05; *p < 0.05; **p < 0.01). H, high expression; HR, hazard ratio; L, low expression; OS, overall survival.

Overall, in the aforementioned experiments, we demonstrated that cold tumors in PAAD express higher levels of *NT5E* and *CD276*. Interestingly, a robust linear relationship between the expression of *NT5E* and *CD276* was noted in PAAD (R = 0.54, p = 2.6e-14) (**Figure 9A**). In addition, levels of *NT5E* and *CD276* were positively associated with tumor grades, but not other clinicopathological characteristics; notably, tumors from elderly patients expressed higher levels of *CD276* (**Figure 9B**). We further examined the links between tumor heterogeneity and the expression of *NT5E* and *CD276*, finding positive correlations between *NT5E* or *CD276* expression and the degree of homologous recombination deficiency and loss of heterozygosity (**Supplementary Figures 7A–C**).

**Figure 10:**
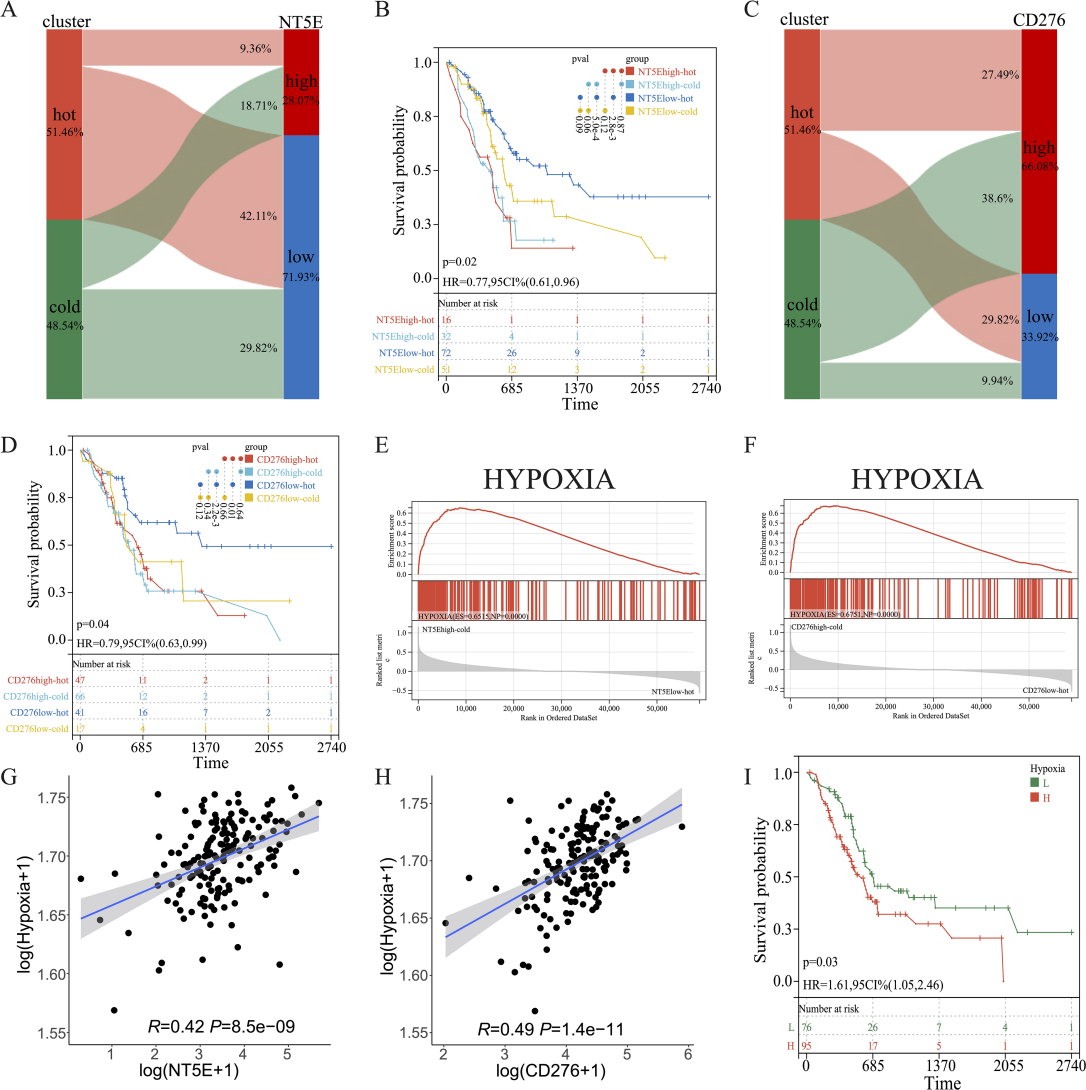
Combined effects of *CD276* and *NT5E* on hypoxia and survival in PAAD. **(A, B)** Sankey diagrams for *NT5E* or *CD276* expression and hot and cold clusters. **(C, D)** KM survival curve of OS between patients in the four indicated groups. **(E, F)** GSEA between patients in the two indicated groups. **(G, H)** Correlation between *NT5E* or *CD276* expression and the hypoxia pathway. **(I)** KM survival curve of OS between patients with high and low hypoxia scores.

KM survival analysis of the TCGA dataset and the other two independent PAAD cohorts revealed that *NT5E* and *CD276* negatively influenced patient survival (**Figures 9C–H**). Subsequently, patients with PAAD were further classified into four groups based on their immune subtypes and *NT5E* expression: *NT5E*high-hot, *NT5E*high-cold, *NT5E*low-hot, and *NT5E*low-cold. Subsequent KM analysis revealed that the *NT5E*low-hot group had the best survival rate among these groups (**Figures 10A, B**). Similarly, the *CD276*low-hot group was associated with a better survival rate compared with that of the *CD276*high-hot, *CD276*high-cold, and *CD276*low-cold groups (**Figures 10C, D**). GSEA between the *NT5E*high-cold and *NT5E*low-hot groups (**Figure 10E**), and between the *CD276*high-cold and *CD276*low-hot groups (**Figure 10F**) revealed that the hypoxia pathway was enriched in the *NT5E*high-cold and *CD276*high-cold groups (**Figures 10E, F**). Positive correlations between the hypoxia pathway and *NT5E* or *CD276* were prominent, as shown in **Figures 10G, H**. As expected, KM survival analysis showed that hypoxia negatively influenced the prognosis of PAAD (**Figure 10I**). Moreover, we identified significant differences in overall survival between the *NT5E*high-*Hypoxia*high and *NT5E*low-*Hypoxia*low groups (**Supplementary Figure 8A**), as well as between the *CD276*high-*Hypoxia*high and *CD276*low-*Hypoxia*low groups (**Supplementary Figure 8B**).

**Figure 11:**
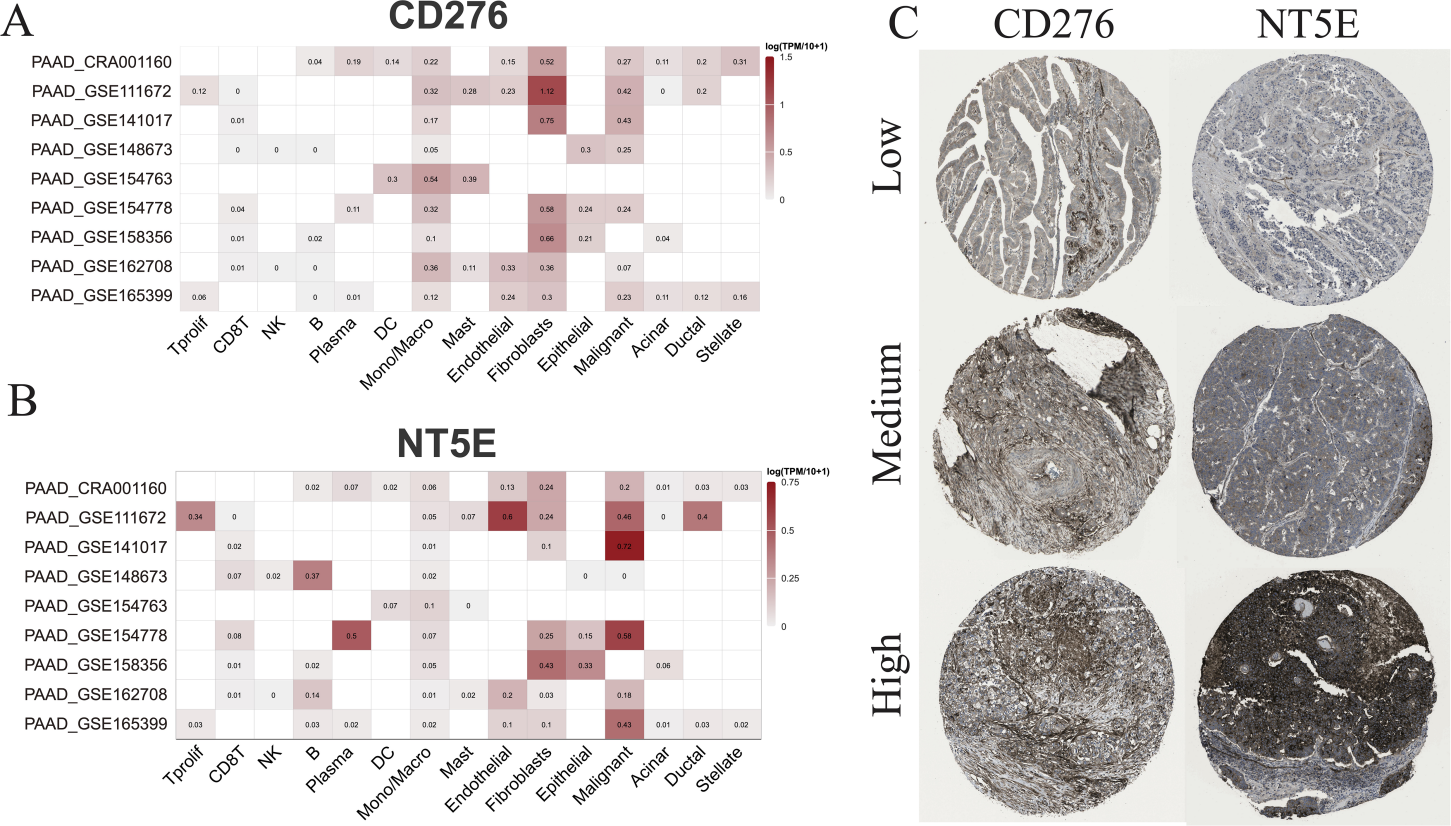
scRNA-seq and IHC analysis. **(A)** The RNA expression levels of CD276 and NT5E across different cell types based on nine scRNA-seq databases. **(B)** The protein expression levels of CD276 and NT5E in tumor tissues are also shown.

Subsequently, multiple single-cell RNA sequencing datasets of PAAD were applied to examine the expression patterns of *NT5E* and *CD276* across different cell populations within the TME. The findings revealed predominant expression of *CD276* and *NT5E* in monocytes/macrophages, fibroblasts, and malignant cells, as illustrated in **Figures 11A, B**. Additionally, protein expression and subcellular localization of CD276 and NT5E were examined using the results of immunohistochemistry analysis of PAAD tissue sections extracted from the HPA database (**Figure 11C**). Next, an mIHC assay was conducted to examine NT5E protein expression and its correlation with hot and cold TME in patients with PAAD, as well as its impact on patient survival. This assay detected NT5E, CD163, and PANCK in the PAAD tissue array, revealing the presence of NT5E+ cells in both tumoral and stromal regions (**Figures 2A–E**). Moreover, a positive correlation was observed between the numbers of NT5E+ cells and CD163+ cells in tumor regions, indicating a potential association between increased NT5E expression and the cold TME in PAAD (**Supplementary Figure 9**). Ultimately, we demonstrated that a greater abundance of NT5E+ cells, irrespective of their spatial distribution, were correlated with diminished patient survival in PAAD, thereby confirming the findings of transcriptomic analysis (**Figure 2H**). The overview image of mIHC staining of the PAAD tissue array is shown in **Supplementary Figure 10**.

### 3.8 Predicting drug sensitivity in hot and cold tumors

**Figure 12:**
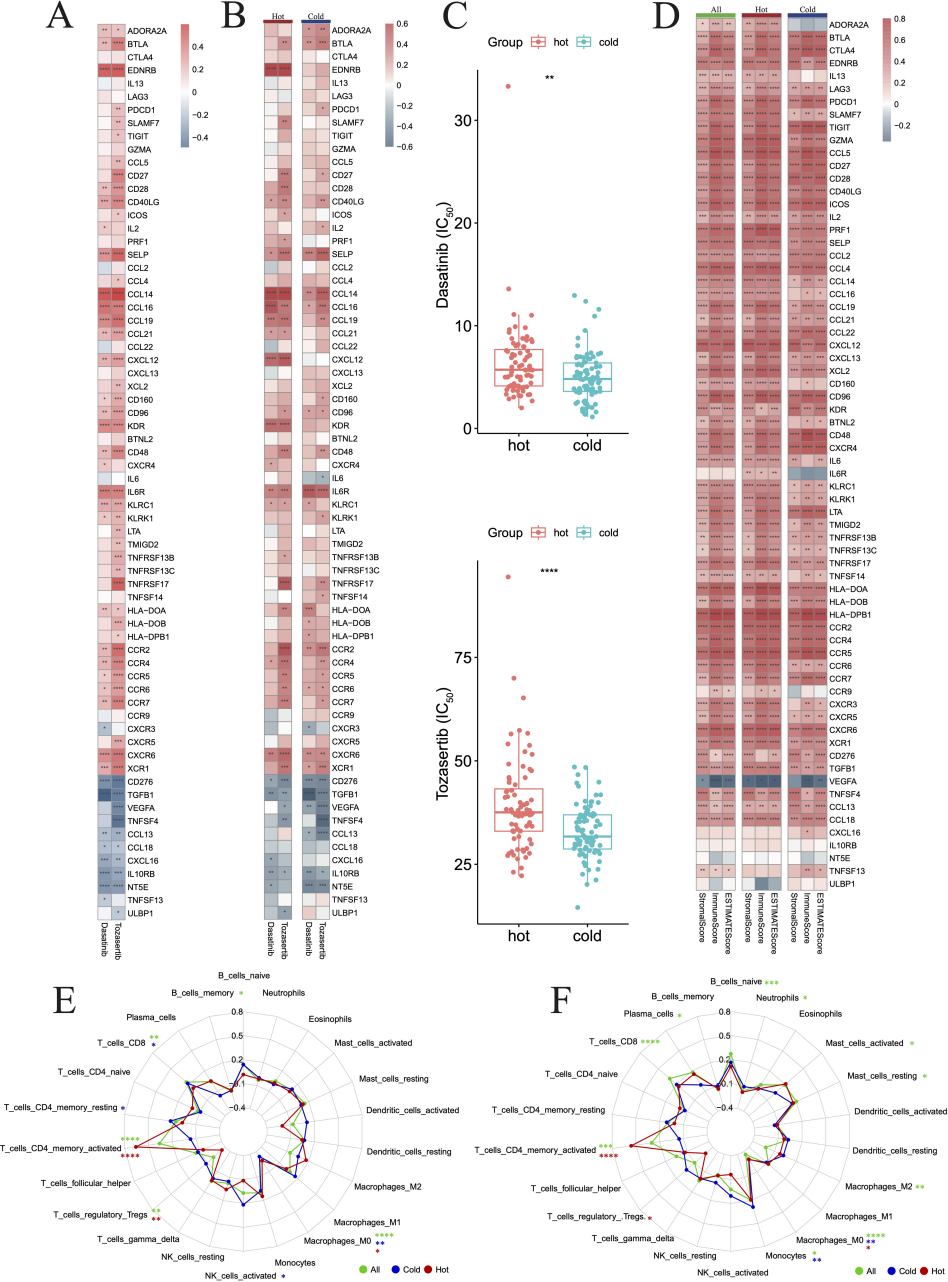
Drug prediction for hot and cold tumors. **(A)** Heatmap showing changes in gene expression induced in all tumors by the two drugs. **(B)** Heatmap showing changes in gene expression induced by the two drugs in hot and cold tumors. **(C)** Box plots showing the difference in drug sensitivity between hot and cold tumors. **(D)** Heatmap showing the correlation between genes and three immune scores across all, hot, and cold tumors. **(E, F)** Radar charts showing the impact of drugs on immune cell infiltration across all, hot, and cold tumors. (*p < 0.05, **p < 0.01, ***p < 0.001, ****p < 0.0001). FIC_50_, half maximal inhibitory concentration.

Herein, we sought to investigate the potential of the DEGs between hot and cold tumors as candidate drug targets in PAAD. Therefore, we employed the oncoPredict to forecast gene correlations with drug responses. Our findings revealed that two drugs (dasatinib and tozasertib) exerted a positive influence on the majority of genes found to be upregulated in hot tumors. Importantly, these agents also negatively influenced most genes that were upregulated in cold tumors, which is a desirable effect (**Figure 12A**). The regulatory mechanisms underlying the effects of these two drugs may differ between hot and cold tumors, as specific genes, such as endothelin receptor type B (*EDNRB*), *CCL14*, and C-X-C motif chemokine ligand 12 (*CXCL12*), were regulated more strongly by both drugs in hot tumors (**Figure 12B**). In addition, both drugs negatively affected the expression of *NT5E* in cold tumors, but minimally affected its levels in hot tumors (**Figure 12B**). The effects of these two drugs were more marked in cold tumors versus hot tumors (**Figure 12C**), as indicated by the lower IC_50_ values. This evidence suggested that these agents could exert more beneficial effects on cold tumors. The beneficial effects of these two drugs were further indicated by the positive correlations between the differentially expressed immune genes and the ImmuneScore, StromaScore, and ESTIMATEScore (three scores used to estimate the extent of tumor immune infiltration) (**Figure 12D**). The effects on various immune cells were further evaluated, and the results indicated that both drugs positively regulated T cell subtypes, particularly CD4+ memory-activated cells in hot tumors (**Figures 12E, F**).

### 3.9 Binding of predicted drug molecules with differentially expression genes

**Figure 13:**
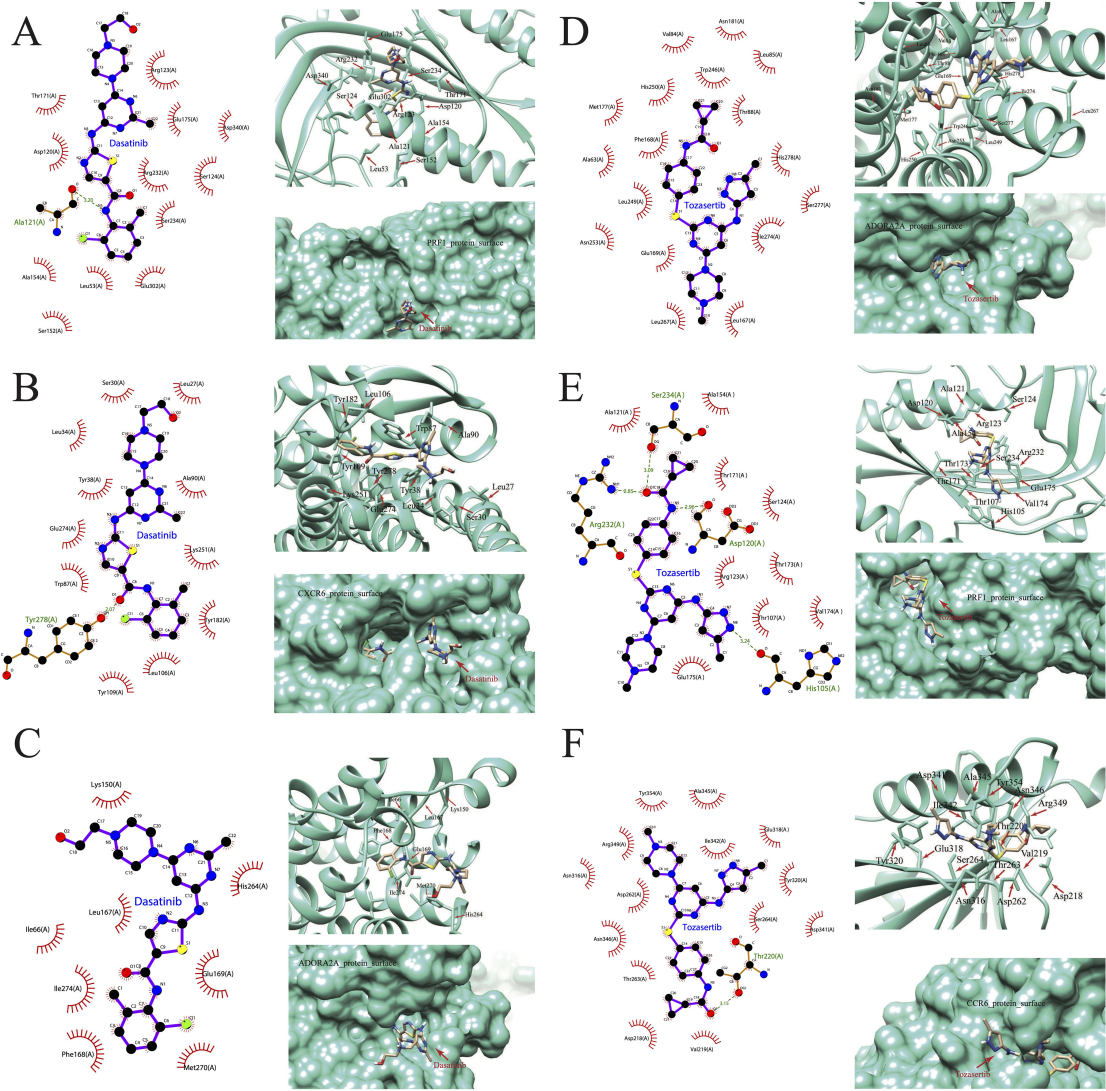
Visualization of drug-protein interactions. **(A)** PRF1, **(B)** CXCR6, and **(C)** ADORA2A bound with dasatinib. **(D)** ADORA2A, **(E)** PRF1, **(F)** CCR6 bound with tozasertib.

We further downloaded the chemical structures of dasatinib and tozasertib from the ZINC15 database. The complete protein structures of the products of the 44 DEGs between hot and cold PAAD were successfully retrieved and preprocessed (**Supplementary Table 2**). Next, we explored the binding potential between proteins and drugs. According to the docking scores (**Supplementary Table 2**), the top three molecules that exhibited the most potent binding affinity with dasatinib were PRF1, CXCR6, and ADORA2A. Further, 2D and 3D molecular visualization of drug-protein interactions demonstrated that dasatinib displayed strong interactions with the PRF1 amino acid residue Ala121 (**Figure 13A**) and the CXCR6 residue Tyr278 (**Figure 13B**). Furthermore, pockets were identified on the surface of the PRF1, CXCR6, and ADORA2A which would allow the formation of a stable complex with dasatinib (**Figures 13A–C**). The top three molecules exhibiting the most robust binding affinity with tozasertib were ADORA2A, PRF1, and CCR6 (**Figures 13D–F**). Tozasertib exhibited robust interactions with the PRF1 residues Ser234, Arg232, and Asp120 amino acids, as well as the Thr220 residue of CCR6 through hydrogen bonds (**Figures 13E, F**). In addition, the pockets on the surface of ADORA2A and CCR6 interacted with tozasertib to form a complex (**Figures 13D, F**).

## 4 Discussion

Overall, this study presented a clinically relevant method for categorizing tumor immune phenotypes. Through this approach, we were able to divide the tumor clusters into immunologically hot and cold categories. We further examined five cancer types that shared some features in terms of hot versus cold tumors, namely BLCA, CESC, PAAD, SARC, and SKCM. In contrast to cold tumors, hot tumors were associated with a higher chance of survival, and displayed higher numbers of CD8+ T cells and activated NK cells, higher scores of T cell proliferation and cytolytic activity, and decreased inflow of M2 macrophages. Additional research revealed that the hot and cold tumors had distinct somatic mutations, gene expression, hallmark pathways, and immune landscapes.

We further determined that the hot/cold immune phenotype was closely associated with immune cell-cell interactions. Specifically, we found that CD8+ T cells were significantly positively correlated with follicular helper T cells and activated NK cells. This finding was consistent with those previously reported by Niogret et al. (53), suggesting that follicular helper T cells assist in reinstating the antitumor activity of exhausted CD8+ T cells, possibly through the production of interleukin-21 (IL-21). Moreover, dendritic cells recruited by intratumoral NK cells can activate CD8+ T cells through the cross-presentation of neoantigens to CD8+ T cells (54). Subsequently, the activated CD8+ T cells and NK cells exert antitumor effects in a concerted and collaborative manner. According to Nicolai et al., stimulator of interferon genes (STING) agonists can effectively promote the activity of NK cells to eliminate tumor cells resistant to CD8+ T cells (55). Evidence from numerous studies have suggested that CD8+ T cells are suppressed by M2 macrophages (56). This is consistent with our findings indicating that M2 macrophages were enriched and inversely associated with CD8+ T cells in cold tumors. M2 macrophages may promote immune evasion and tumor progression through multiple mechanisms. For example, M2 macrophage-derived extracellular vesicles may trigger CD8+ T cell exhaustion, and thereby promote tumor progression in hepatocellular carcinoma (57). Additionally, M2 macrophages can inhibit T and NK cell-mediated antitumor activity by secreting an array of immunosuppressive cytokines, including IL-10 and transforming growth factor-β (TGF-β), or by expressing co-inhibitory ligands that directly inhibit T cell activation (58, 59).

A previous study has used cancer immunograms to visualize the overall immune landscape of the TME (60), and aids in identifying critical immunosuppressive factors and selecting appropriate targeting strategies for immunotherapy. Our research presents an immunogram through a radar plot that employs seven antitumoral parameters and five protumoral parameters to illustrate the immunological characteristics of each tumor. Previous studies have suggested several immunograms for the personalized treatment of breast cancer (61), lung cancer (62), hepatocellular carcinoma (63), and urothelial cancer (64). Importantly, our immunogram includes parameters that were not previously used in combination, and have identified critical immunological factors involved in the establishment of hot or cold tumors. However, as most immunograms have not been tested in clinical settings, clinical trials are necessary to evaluate the potential of these models, including the one presented here, in guiding individualized cancer treatment.

*NT5E* and *CD276* were found to be upregulated in cold tumors, suggesting that they may play key roles in inhibiting CD8+ T cells and promoting M2 macrophages. Recent studies have further demonstrated that NT5E overexpression hinders CD8+T cell recruitment and directly inhibits T cell antitumor activity in several preclinical cancer models (65, 66). It has also been reported that tumor necrosis factor-α (TNF-α) and interferon-α (IFN-α) induce NT5E expression in mesenchymal stem cells, thereby promoting the polarization of anti-inflammatory M2 macrophages (67); however, another study showed that NT5E is not required for M2 macrophage polarization (68). As such, the impact of NT5E on M2 macrophages warrants further investigation. It has further been shown that CD276 (B7-H3) increases the ability of colorectal cancer cell lines to resist apoptosis by activating the Janus kinase 2-signal transducer and activator of transcription 3 (JAK2-STAT3) pathway (69). CD276 may further stimulate the nuclear factor-κB (NF-κB) pathway and enhance angiogenesis in colorectal cancer (70). Nevertheless, the role of CD276 in tumorigenesis and immune responses remains unclear in several types of cancer.

Overall, in the present study, we found that cold tumors were associated with shorter survival. On this basis, we questioned whether *NT5E* and *CD276*, which are upregulated in cold tumors and are linked to poor survival, could be used as biomarkers and potential targets to convert cold tumors into hot tumors. Our analysis of the roles of these two genes in PAAD was further prompted by their linear co-expression patterns, which suggested that they may be functionally connected. GSEA revealed that the hypoxia pathway was enriched in the *NT5E*high and *CD276*high tumors. Indeed*, NT5E* has been reported as a hypoxia-responsive gene (71), and a previous pan-cancer study revealed that NT5E is overexpressed and correlated with a worse prognosis in several cancer types, including PAAD (72). Previous studies have reported that CD276 regulates hypoxia by stimulating aberrant angiogenesis, which elevates hypoxemia within the TME, thereby hindering the entry of CD8+ T cells (73). As such, the regulation of NT5E and CD276 on hypoxia may be responsible for establishing the cold immune state in PAAD. Studies have also suggested that targeting NT5E has therapeutic effects in some preclinical cancer models (74, 75). Moreover, a phase I clinical trial using a small-molecule NT5E inhibitor in patients with pancreatic cancer is ongoing; early results are promising, with an overall response rate of 41%. In our cohort, NT5E was abundant in both the tumoral and stromal regions of PAAD tissues. Moreover, the levels of NT5E were associated with poor patient survival. These findings indicate that examining the expression of NT5E protein through traditional IHC staining in clinical practice may help to select patients with cold tumors for NT5E-targeted therapy. We further speculate that targeting CD276 and NT5E in combination may improve treatment outcomes.

The current study has some limitations that may impact the interpretation of our results. First, our analyses centered on five cancer types, and we primarily focused on the identification of biomarkers in PAAD. Further, we only conducted some experimental validation using the PAAD cohort. Therefore, larger clinical cohorts, ideally comprising multiple cancer types, are required to further test the usefulness and credibility of potential biomarkers, and more functional studies are needed to better understand the role of these molecules. In addition, our study only included a limited number of immune regulatory genes, meaning there may be other, unidentified crucial genes involved in the development of hot/cold TME. Nevertheless, we hope that the platform established in the current analysis could help explore more genes and identify critical biomarkers, candidate drugs, and drug targets for cancer immunotherapy.

## 5 Conclusion

In summary, our research developed a tumor classification approach that utilizes parameters linked to immune cell infiltration and immunological traits in the TME. This classification allowed for the differentiation of hot and cold tumors, as well as the prediction of patient survival. The tumor and immune phenotypes varied significantly between hot and cold tumors. The increased expression of some hub genes in cold tumors suggests a potential role of these molecules in treating cold tumors or inducing cold-to-hot tumor transition. Moreover, dasatinib and tozasertib may be useful in modulating the TME in cold tumors, and thereby promoting anticancer immune response.

## Data Availability

The original contributions presented in the study are included in the article/**Supplementary Material**. Further inquiries can be directed to the corresponding author/s.
