# Supplementary material for: Immune regulatory genes impact the hot/cold tumor microenvironment, affecting cancer treatment and patient outcomes
Source: Front Immunol. 2025 Jan 22;15:1382842. doi: 10.3389/fimmu.2024.1382842 (PMC11794490; doi:10.3389/fimmu.2024.1382842)
Supplement: Supplementary file 1 [file DataSheet1.docx]

**Supplemental figures and figure legends:**

**Supplemental Figure 1. Heatmap showing immune cell infiltrations and immunological feature scores.** (A-H) Heatmaps showing immune cell infiltrations and immunological feature scores in hot and cold tumors, and clinical features of BLCA (A), CESC (B), PAAD (C), SARC (D), SKCM (E) KIRP (F), LGG (G), and THYM (H). *, **, ***, and **** represent significant difference between hot and cold tumors. (+) represents higher values in hot tumors. (-) represents higher values in cold tumors (ns: p > 0.05, *: p < 0.05, **: p < 0.01, ***: p < 0.001, ****: p < 0.0001).


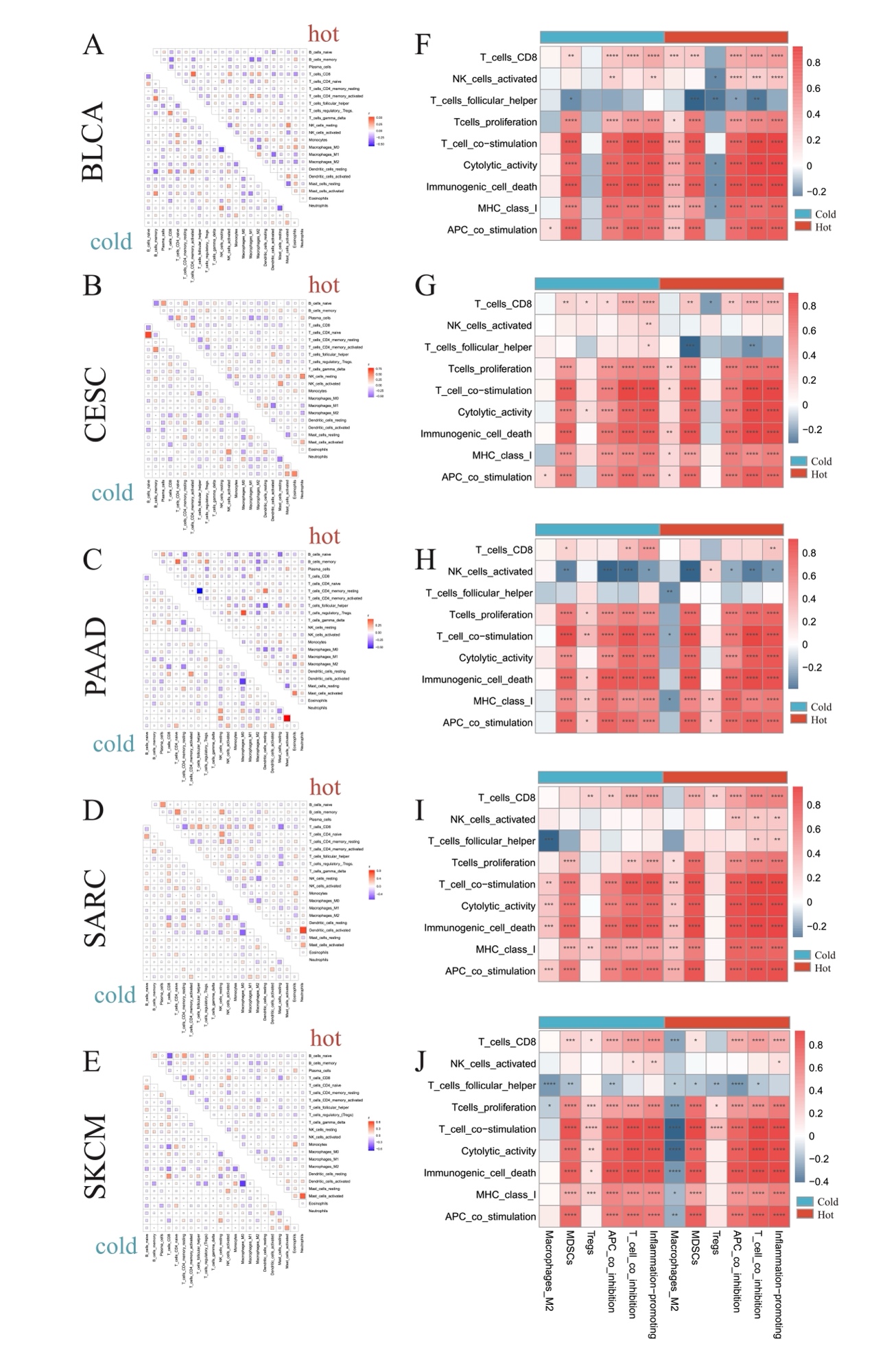


**Supplemental Figure 2. The correlations between 22 immune cell infiltrations in BLCA (A), CESC (B), PAAD (C), SARC (D), and SKCM (E). Correlations between key immune stimulatory factors and immunosuppressive factors in hot and cold tumors of BLCA (F), CESC (G), PAAD (H), SARC (I), and SKCM (J).**


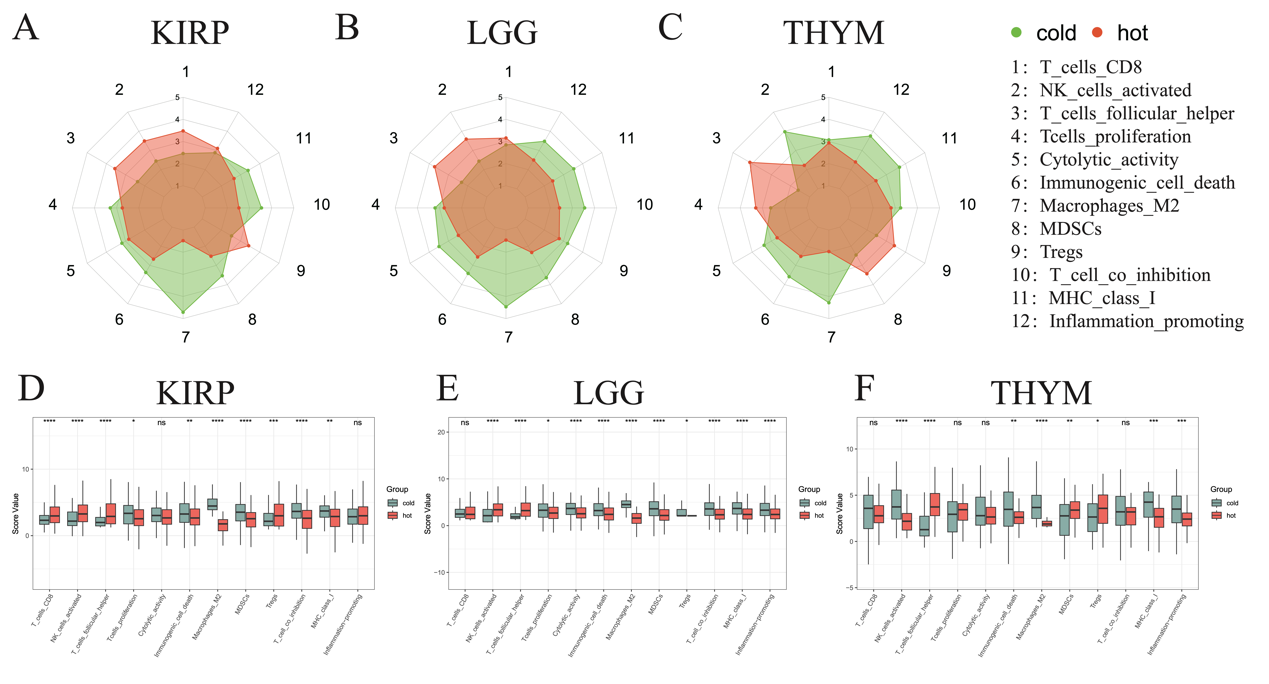


**Supplemental Figure 3. Differences in the 12 main characteristics of cold and hot tumors.** Radar chart showing 12 main characteristics of cold and hot tumors in KIRP (A), LGG (B), and THYM (C). Histogram showing 12 main characteristics of cold and hot tumors in KIRP (D), LGG (E), and THYM (F) (ns: p > 0.05, *: p < 0.05, **: p < 0.01, ***: p < 0.001, ****: p < 0.0001).


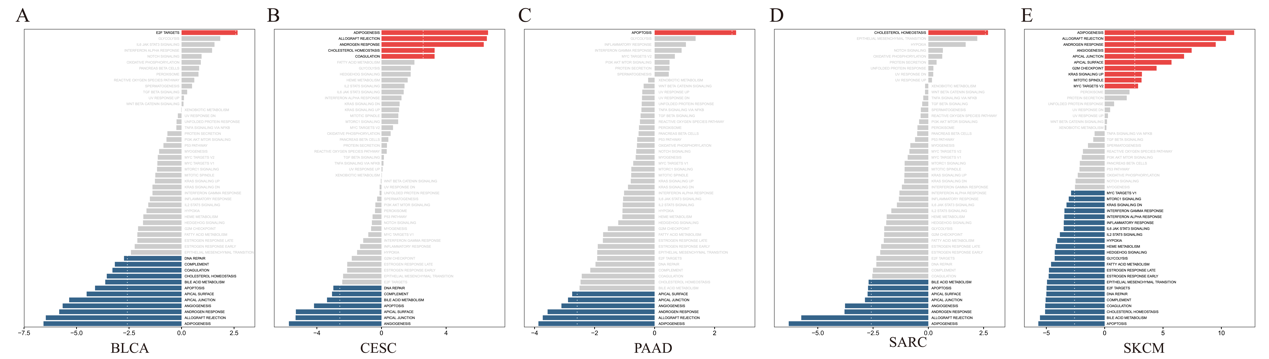


**Supplemental Figure 4. GSVA analysis of hallmark pathways.** GSVA analysis of hallmark pathways in BLCA (A), CESC (B), PAAD (C), SARC (D), and SKCM (E).

**Supplemental Figure 5. GSVA analysis of KEGG pathways.** GSVA analysis of the KEGG pathways in BLCA (A), CESC (B), PAAD (C), SARC (D), and SKCM (E). (F, G) Veen plots showing the different KEGG pathways between hot and cold tumors in BLCA, CESC, PAAD, SARC, and SKCM ( F: up-expression in hot tumors, G: down-expression in hot tumors). (H) The correlations between 23 genes and 6 pathways in BLCA, CESC, PAAD, SARC, and SKCM (ns: p > 0.05, *: p < 0.05, **: p < 0.01, ***: p < 0.001, ****: p < 0.0001).

**Supplemental Figure 6. The correlations between immune regulator genes and immune cell infiltrations** (ns: p > 0.05, *: p < 0.05, **: p < 0.01, ***: p < 0.001, ****: p < 0.0001).


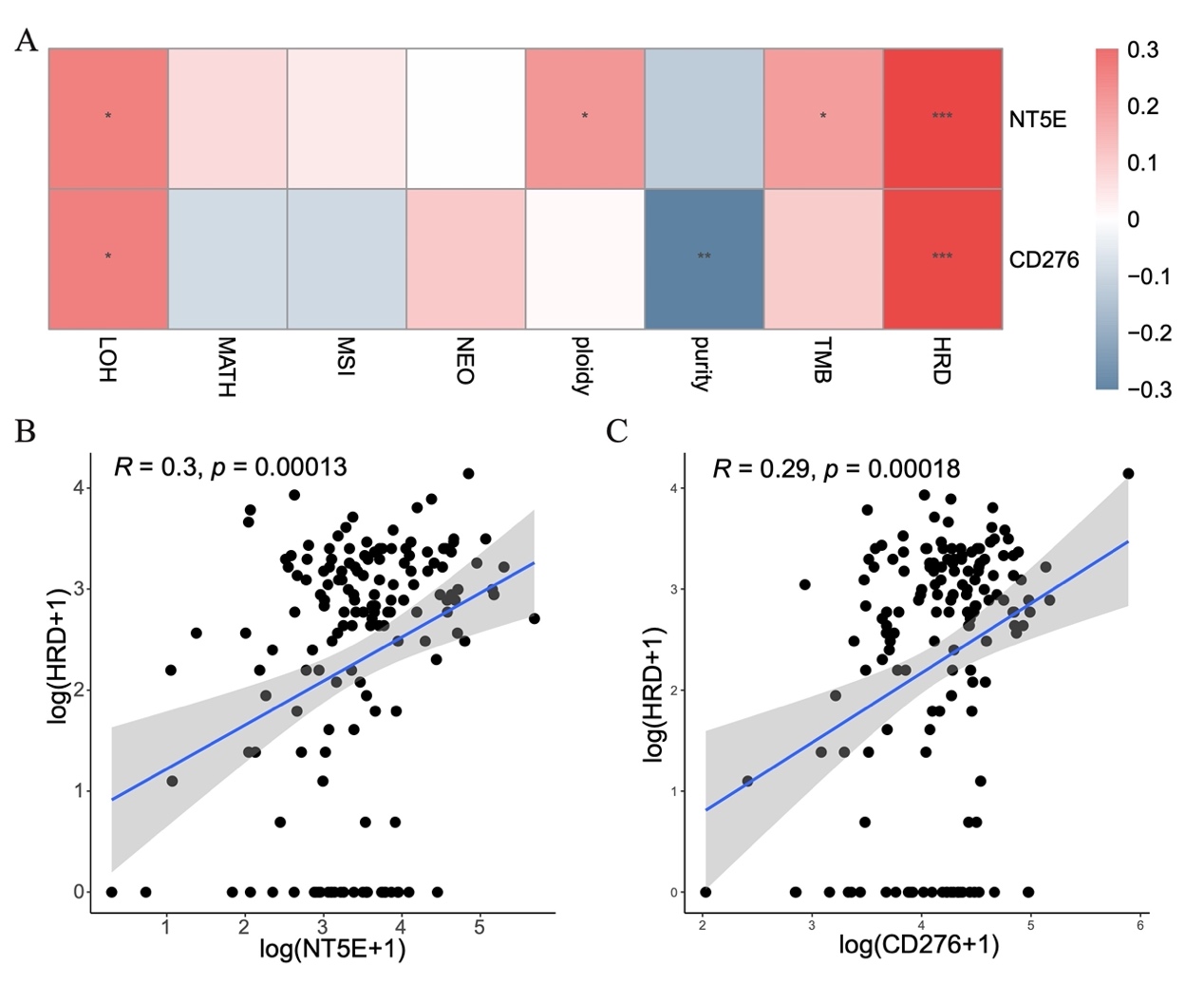


**Supplemental Figure 7. The correlations between genomic heterogeneity and gene expression.** (A) Heatmap showing the correlations between values of genomic heterogeneity and the levels of NT5E and CD276. (B, C) The linear graphs showing the correlations between values of HRD and the expressions of NT5E and CD276 (ns: p > 0.05, *: p < 0.05, **: p < 0.01, ***: p < 0.001, ****: p < 0.0001).

**Supplemental Figure 8. KM survival analysis. (**A, B) KM survival curve of OS of patients in the four groups (A: *NT5E*high-*Hypoxia*high, *NT5E*high-*Hypoxia*low, *NT5E*low-*Hypoxia*high, and *NT5E*low-*Hypoxia*low; B: *CD276*high-*Hypoxia*high, *CD276*high-*Hypoxia*low, *CD276*low-*Hypoxia*high, and *CD276*low-*Hypoxia*low).


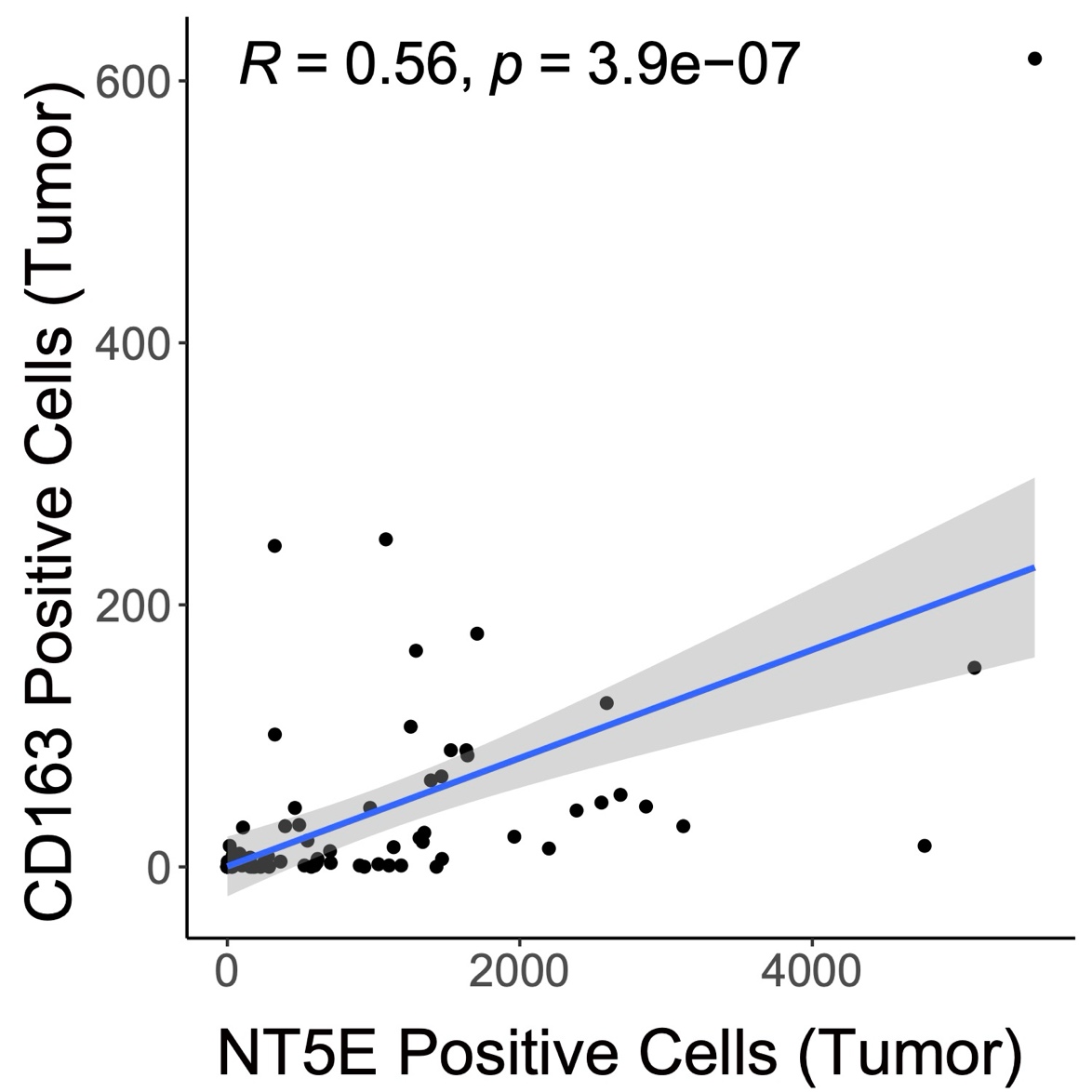


**Supplemental Figure 9. The correlation between the numbers of tumoral NT5E+ cells and tumoral CD163+ cells**

**Supplemental Table 1.** The correlation coefficients between immune genes and four types of immune cells in PAAD, including CD8+ T cells, M2-type macrophages, activated NK cells, and follicular helper T cells.

| **GENES** | **T_cells_CD8** | **T_cells_follicular_helper** | **NK_cells_activated** | **Macrophages_M2** |
| --- | --- | --- | --- | --- |
| ADORA2A | 0.134184499 | 0.134119624 | -0.006114057 | -0.199998112 |
| ARG1 | -0.003011667 | 0.114270073 | 0.210009294 | 0.011452553 |
| BTLA | 0.139333614 | 0.164775764 | -0.098954621 | -0.353670807 |
| CD274 | 0.090946458 | -0.141313351 | -0.231243734 | 0.128113759 |
| CD276 | -0.186651081 | 0.011857369 | -0.160753905 | 0.196222733 |
| CTLA4 | 0.230474829 | 0.159672451 | -0.162995843 | -0.24679798 |
| EDNRB | 0.236891827 | -0.112458937 | -0.142033878 | -0.122248065 |
| HAVCR2 | 0.128775463 | -0.218926123 | -0.282448965 | 0.183062749 |
| IDO1 | 0.162339211 | -0.026118772 | 0.065142462 | -0.022923089 |
| IL10 | 0.145792603 | -0.036441965 | -0.217412774 | 0.059246424 |
| IL13 | 0.15226075 | 0.088859436 | 0.041829371 | -0.142526874 |
| IL4 | 0.068789743 | 0.011280059 | -0.009821099 | -0.040617222 |
| KIR2DL1 | 0.17650485 | -0.087370993 | -0.047418752 | 0.046054391 |
| KIR2DL3 | 0.141939381 | -0.09981373 | -0.100762843 | 0.022744178 |
| LAG3 | 0.226913328 | 0.151182458 | 0.058458897 | -0.103791172 |
| PDCD1 | 0.265895981 | 0.263341624 | -0.124197484 | -0.32225073 |
| SLAMF7 | 0.23608253 | -0.039543556 | -0.224932722 | -0.145196191 |
| TGFB1 | -0.076876491 | 0.079511139 | -0.255988612 | 0.097201001 |
| TIGIT | 0.236465226 | 0.152379392 | -0.133567135 | -0.307862636 |
| VEGFA | -0.246885628 | 0.001086432 | -0.066696704 | 0.0087558 |
| VEGFB | 0.065857519 | 0.207001033 | 0.059144721 | 0.05822253 |
| C10orf54 | 0.048706451 | -0.008708782 | -0.172250142 | -0.057653589 |
| VTCN1 | 0.225227992 | 0.064727259 | -0.122046973 | -0.073733867 |
| IL12A | 0.208671615 | 0.079337416 | -0.16831802 | -0.086901743 |
| GZMA | 0.408675919 | 0.04145099 | -0.101333754 | -0.280241677 |
| BTN3A1 | 0.223260287 | -0.060569233 | -0.212289579 | -0.186602456 |
| BTN3A2 | 0.185755584 | -0.092386382 | -0.290331305 | -0.127600337 |
| CCL5 | 0.486249525 | 0.080342732 | -0.107660109 | -0.300186456 |
| CD27 | 0.200702192 | 0.21639529 | -0.130211825 | -0.364357828 |
| CD28 | 0.22662871 | 0.077855642 | -0.181227032 | -0.288802363 |
| CD40 | 0.097180815 | 0.099178295 | -0.22416942 | -0.092512291 |
| CD40LG | 0.219825723 | 0.112080544 | -0.128474836 | -0.318226216 |
| CD70 | 0.051740646 | 0.170901584 | -0.111749158 | -0.049907375 |
| CD80 | 0.02400139 | -0.066308832 | -0.233753575 | 0.066902368 |
| CX3CL1 | -0.051698565 | 0.120948409 | -0.006745027 | 0.015420325 |
| CXCL10 | 0.15151462 | -0.056028376 | -0.050398711 | 0.031671273 |
| CXCL9 | 0.159247434 | -0.084049409 | -0.085315164 | -0.032293157 |
| ENTPD1 | 0.147041628 | -0.145375503 | -0.324621041 | 0.025331325 |
| HMGB1 | -0.002773253 | -0.035210937 | -0.169911929 | 0.03664579 |
| ICAM1 | -0.012449906 | -0.037527842 | -0.254736843 | -0.025290249 |
| ICOS | 0.259715462 | 0.140334245 | -0.150549506 | -0.281344443 |
| ICOSLG | 0.048172325 | 0.094918902 | 0.077256459 | -0.06967731 |
| IFNA1 | -0.03972851 | -0.006775214 | 0.066272076 | -0.064457416 |
| IFNA2 | -0.134675164 | -0.133350099 | 0.037920246 | -0.052824946 |
| IFNG | 0.210544583 | -0.003668934 | -0.039390607 | -0.118841837 |
| IL1A | -0.149139392 | 0.185239309 | -0.084346234 | -0.066636528 |
| IL1B | 0.090367854 | -0.041206016 | -0.147879591 | -0.144878738 |
| IL2 | 0.291241213 | 0.056427802 | -0.085578815 | -0.201280125 |
| IL2RA | 0.033024622 | -0.159410944 | -0.31879374 | 0.11655953 |
| ITGB2 | -0.022830944 | -0.158929894 | -0.316701884 | 0.091518565 |
| PRF1 | 0.325924099 | 0.026596113 | -0.153314969 | -0.12776622 |
| SELP | 0.434453411 | 0.008082303 | -0.135931577 | -0.227341978 |
| TLR4 | 0.097228234 | -0.296176722 | -0.250951743 | 0.11530376 |
| TNF | -0.022857348 | 0.142119377 | 0.025992524 | -0.084866995 |
| TNFRSF14 | -0.046061707 | 0.099311806 | 0.090610823 | -0.129052067 |
| TNFRSF18 | 0.011098059 | 0.32540836 | -0.072833562 | -0.167049088 |
| TNFRSF4 | 0.017112297 | 0.249720147 | -0.027425974 | -0.174472827 |
| TNFRSF9 | 0.043031936 | 0.082219115 | -0.150629712 | -0.165851446 |
| TNFSF4 | -0.11353618 | -0.155342085 | -0.241949262 | 0.209394897 |
| TNFSF9 | -0.11943793 | 0.038531755 | 0.028440396 | 0.000613891 |
| CCL1 | 0.020356329 | 0.005272993 | -0.017037283 | -0.000228701 |
| CCL2 | 0.260798052 | 0.084521842 | -0.062759652 | -0.173649733 |
| CCL3 | 0.067340716 | 0.125222058 | 0.038786012 | 0.032147853 |
| CCL4 | 0.402283391 | 0.150523722 | -0.036038614 | -0.178742377 |
| CCL7 | -0.085336347 | 0.050256266 | -0.125425017 | 0.176557508 |
| CCL8 | 0.166758366 | 0.085111513 | -0.082467826 | 0.000156001 |
| CCL11 | 0.089016959 | 0.007481534 | -0.08508125 | 0.016497359 |
| CCL13 | -0.071926275 | -0.105922653 | -0.277529425 | 0.338028752 |
| CCL14 | 0.356271445 | 0.020397026 | -0.093847273 | -0.206522845 |
| CCL15 | 0.018735225 | -0.046550517 | 0.195159388 | -0.06246584 |
| CCL16 | 0.321190779 | -0.047821476 | -0.122678177 | -0.107559746 |
| CCL17 | 0.139507928 | -0.071955533 | -0.201422894 | -0.035375755 |
| CCL18 | -0.056916443 | -0.113663573 | -0.148505787 | 0.283371764 |
| CCL19 | 0.246967644 | 0.200153189 | -0.078985364 | -0.382795074 |
| CCL20 | -0.12911359 | 0.091699305 | -0.057929682 | -0.065607288 |
| CCL21 | 0.127465136 | 0.139631281 | -0.086086434 | -0.245491838 |
| CCL22 | 0.193044397 | 0.04880616 | -0.1683779 | -0.230829177 |
| CCL23 | 0.152355314 | -0.015311829 | -0.112582605 | 0.032361307 |
| CCL24 | -0.120418485 | -0.160643891 | 0.316042434 | -0.083232056 |
| CCL25 | 0.030328865 | 0.017702663 | 0.048705825 | -0.007325688 |
| CCL26 | -0.067324007 | -0.000257718 | 0.131825846 | -0.004955955 |
| CCL27 | 0.015861961 | 0.125626483 | 0.083194324 | 0.020581323 |
| CCL28 | 0.08369004 | -0.03096081 | -0.156996759 | -0.029646304 |
| CXCL1 | 0.12395022 | 0.1894001 | -0.105586178 | -0.218201823 |
| CXCL2 | 0.20874996 | 0.146173885 | -0.13994646 | -0.220486059 |
| CXCL3 | 0.060453446 | 0.117867405 | -0.094475597 | -0.188169766 |
| CXCL5 | -0.142935759 | -0.083564167 | 0.003612692 | 0.066642485 |
| CXCL6 | 0.167993492 | -0.021579484 | -0.112072949 | 0.002293285 |
| CXCL8 | -0.116338856 | -0.035393298 | -0.128407826 | 0.059333759 |
| CXCL11 | 0.164139966 | -0.089689012 | -0.009060185 | -0.035507796 |
| CXCL12 | 0.281788667 | -0.013768923 | -0.202476063 | -0.119484992 |
| CXCL13 | 0.106710157 | 0.254332498 | -0.069973767 | -0.36280026 |
| CXCL14 | 0.00011843 | -0.093709013 | -0.113955657 | 0.008780631 |
| CXCL16 | -0.176396102 | -0.256823497 | -0.056420247 | 0.166808171 |
| CXCL17 | -0.139296789 | -0.043689609 | -0.040837664 | 0.014575851 |
| XCL1 | 0.172730045 | 0.084551379 | -0.135848383 | -0.089306416 |
| XCL2 | 0.425639766 | 0.114125696 | -0.067106397 | -0.260808673 |
| CD160 | 0.299428973 | 0.044248859 | -0.057628136 | -0.137037545 |
| CD244 | 0.206612938 | -0.093142074 | -0.152367521 | -0.142583791 |
| CD96 | 0.352301983 | 0.057642994 | -0.186678347 | -0.30629579 |
| CSF1R | 0.14572179 | -0.235842321 | -0.295230551 | 0.136416171 |
| IL10RB | -0.14783282 | -0.126119218 | -0.151013357 | -0.014032513 |
| KDR | 0.18349831 | -0.079612398 | -0.06019798 | -0.034028574 |
| LGALS9 | -0.013931723 | 0.024788767 | 0.026656596 | -0.105268639 |
| PDCD1LG2 | 0.086480556 | -0.134504918 | -0.254699944 | 0.105110035 |
| PVRL2 | -0.139587312 | 0.081413112 | 0.096497252 | 0.02009611 |
| TGFBR1 | 0.024136457 | -0.186843756 | -0.280412845 | 0.148787704 |
| BTNL2 | 0.126523025 | 0.159068665 | -0.10505064 | -0.307170987 |
| CD48 | 0.201362337 | 0.138999268 | -0.110325914 | -0.335543225 |
| CD86 | 0.099403189 | -0.181816278 | -0.298478643 | 0.129330812 |
| CXCR4 | 0.159303892 | 0.115184311 | -0.165816264 | -0.281809436 |
| HHLA2 | -0.111718318 | -0.177287604 | -0.079353104 | 0.038502192 |
| IL6 | 0.206557271 | 0.084138258 | -0.110396102 | -0.125405248 |
| IL6R | 0.14183561 | -0.055136282 | -0.027283348 | -0.025172517 |
| KLRC1 | 0.436884064 | -0.071046111 | -0.022444069 | -0.145639526 |
| KLRK1 | 0.421094894 | 0.094888533 | -0.135258419 | -0.295998485 |
| LTA | 0.159606133 | 0.213304496 | -0.054682177 | -0.368865304 |
| MICB | -0.079532521 | -0.033499289 | -0.107864385 | -0.047766202 |
| NT5E | -0.235542615 | -0.156030982 | -0.123342755 | 0.208722563 |
| PVR | -0.107654997 | 0.060053871 | 0.144643899 | 0.184662641 |
| RAET1E | -0.013452133 | -0.045351228 | -0.190704305 | 0.042764183 |
| TMEM173 | -0.00279947 | 0.005394757 | -0.164919497 | -0.035095313 |
| TMIGD2 | 0.196487229 | 0.126575005 | -0.074510081 | -0.275481844 |
| TNFRSF13B | 0.085356002 | 0.168534106 | -0.05162637 | -0.327094206 |
| TNFRSF13C | 0.086657212 | 0.19530748 | -0.067825824 | -0.334826748 |
| TNFRSF17 | 0.189911749 | 0.197163822 | -0.159662254 | -0.350293891 |
| TNFRSF25 | -0.05529947 | 0.182351513 | -0.052736187 | -0.155706209 |
| TNFRSF8 | 0.192000845 | 0.013968188 | -0.171262711 | -0.08443879 |
| TNFSF13 | -0.077779532 | -0.23961049 | -0.262061522 | 0.11989321 |
| TNFSF13B | 0.217674142 | -0.242440951 | -0.296685265 | -0.006832809 |
| TNFSF14 | 0.117686262 | 0.121056524 | -0.030079377 | -0.128648601 |
| TNFSF15 | 0.043583599 | -0.013040721 | -0.121748087 | -0.084449241 |
| TNFSF18 | 0.069212127 | -0.209837314 | -0.109450538 | -0.034585806 |
| ULBP1 | -0.147587062 | -0.077066788 | -0.113234685 | 0.116624617 |
| B2M | 0.055669601 | -0.094831832 | -0.150255215 | 0.030505983 |
| HLA-A | -0.097120789 | 0.013393554 | 0.052043231 | -0.094905938 |
| HLA-B | -0.018874739 | -0.025237232 | -0.148631674 | -0.097572275 |
| HLA-C | -0.038334061 | -0.006134851 | -0.04559595 | -0.125520938 |
| HLA-DMA | 0.254164846 | -0.092086499 | -0.218318678 | -0.066774559 |
| HLA-DMB | 0.226977739 | -0.094195386 | -0.24369023 | -0.05038037 |
| HLA-DOA | 0.246176178 | -0.056587935 | -0.263792337 | -0.121312208 |
| HLA-DOB | 0.11933993 | 0.210665091 | -0.074661271 | -0.362599796 |
| HLA-DPA1 | 0.231648371 | -0.138719954 | -0.267041265 | -0.006291289 |
| HLA-DPB1 | 0.302302783 | -0.07271183 | -0.233934671 | -0.096322771 |
| HLA-DQA1 | 0.207590246 | -0.132200639 | -0.262091651 | 0.005254409 |
| HLA-DQA2 | 0.139093167 | -0.018052361 | -0.137999802 | -0.116731453 |
| HLA-DQB1 | 0.20425677 | -0.12205841 | -0.258972918 | 0.025223162 |
| HLA-DRA | 0.222954735 | -0.155153359 | -0.275035154 | -0.006852607 |
| HLA-DRB1 | 0.182414463 | -0.154875461 | -0.258371952 | 0.059749647 |
| HLA-E | 0.18943062 | -0.05112693 | -0.213458351 | -0.205248928 |
| HLA-F | -0.004295317 | 0.046102231 | -0.006543595 | -0.197939918 |
| HLA-G | -0.165966918 | -0.110296024 | -0.043909385 | 0.174558123 |
| TAP1 | 0.04309005 | -0.02145491 | -0.06195983 | -0.010594776 |
| TAP2 | 0.090656431 | 0.042010383 | -0.093207964 | -0.076256581 |
| TAPBP | -0.130882081 | 0.041443204 | 0.038344436 | -0.098973275 |
| CCR1 | 0.170344205 | -0.19912185 | -0.300267967 | 0.095075253 |
| CCR2 | 0.308614276 | -0.168002774 | -0.21814404 | -0.11809027 |
| CCR3 | 0.038739227 | 0.130429749 | -0.175022836 | -0.063228771 |
| CCR4 | 0.287625084 | 0.023947284 | -0.192798083 | -0.279834517 |
| CCR5 | 0.379886136 | -0.084739762 | -0.189547815 | -0.167715908 |
| CCR6 | 0.075561072 | -0.046913589 | -0.127692114 | -0.276892032 |
| CCR7 | 0.138817444 | 0.156475175 | -0.095990441 | -0.338370186 |
| CCR8 | 0.15542449 | -0.130401309 | -0.231399113 | -0.024368061 |
| CCR9 | 0.048127375 | -0.116209888 | -0.050675733 | -0.122678395 |
| CCR10 | 0.078389325 | 0.167493616 | 0.196810139 | -0.05846489 |
| CXCR1 | 0.046831177 | -0.163416863 | -0.152950927 | 0.005969768 |
| CXCR2 | 0.045846058 | -0.207532216 | -0.135600243 | 0.045776787 |
| CXCR3 | 0.208341328 | 0.107146706 | -0.133966359 | -0.212620413 |
| CXCR5 | 0.081872536 | 0.179757971 | -0.036605535 | -0.376934747 |
| CXCR6 | 0.479667274 | -0.017376243 | -0.189836763 | -0.219669908 |
| XCR1 | 0.279947085 | -0.091369785 | -0.139677323 | -0.253163122 |
| CX3CR1 | 0.167396027 | -0.135917981 | -0.059883049 | -0.042054746 |

Supplemental Table 2. Docking scores between drugs and proteins corresponding to the DEGs.

|  | Dasatinib | Tozasertib |
| --- | --- | --- |
| ADORA2A | -31.364058 | -37.7179 |
| PRF1 | -33.021946 | -35.2295 |
| CCR6 | -31.297157 | -33.9426 |
| XCR1 | -25.972181 | -33.3017 |
| CXCR6 | -31.985685 | -32.9694 |
| IL10RB | -29.410662 | -32.7581 |
| CXCR5 | -28.331308 | -32.2244 |
| TNFSF4 | -23.115484 | -31.3471 |
| NT5E | -26.999372 | -31.3273 |
| XCL2 | -22.710541 | -30.9328 |
| CTLA4 | -29.632147 | -30.8457 |
| VEGFA | -23.7223 | -29.5924 |
| CCL19 | -26.519627 | -29.5223 |
| TNFRSF17 | -21.363365 | -28.8312 |
| IL13 | -24.572309 | -28.8086 |
| CD48 | -24.5828 | -28.1883 |
| BTNL2 | -21.630381 | -28.1355 |
| CXCL12 | -17.816069 | -28.0006 |
| CCL21 | -21.331507 | -27.9648 |
| CD28 | -21.831301 | -27.526 |
| ULBP1 | -17.382057 | -26.8287 |
| KLRK1 | -18.246443 | -26.5589 |
| IL6 | -24.110189 | -26.4775 |
| KLRC1 | -22.992809 | -26.2311 |
| CCL22 | -14.314922 | -26.1064 |
| CXCL16 | -22.252195 | -25.7257 |
| ICOS | -13.898898 | -25.0926 |
| CXCR4 | -16.629736 | -24.4436 |
| CCL5 | 0 | -24.2399 |
| LTA | -8.76201 | -24.1619 |
| CD276 | -21.331551 | -24.046 |
| CCL14 | -15.431384 | -23.9224 |
| TGFB1 | -17.064663 | -23.5813 |
| CD40LG | -19.220009 | -23.0428 |
| BTLA | -18.949467 | -22.8359 |
| CD27 | -14.648325 | -22.7233 |
| TMIGD2 | 0 | -21.9746 |
| PDCD1 | -17.791615 | -20.9067 |
| CCL2 | -16.495134 | -19.2183 |
| CCL4 | -16.495134 | -19.2183 |
| CCR2 | -7.656692 | -13.5028 |
| TNFRSF13B | 0 | -11.6227 |
| SELP | 0 | -8.47214 |
| TNFSF13 | -0.001234 | -0.00084 |
