# Supplementary figures and images for: Immune regulatory genes impact the hot/cold tumor microenvironment, affecting cancer treatment and patient outcomes
Source: Front Immunol. 2025 Jan 22;15:1382842. doi: 10.3389/fimmu.2024.1382842 (PMC11794490; doi:10.3389/fimmu.2024.1382842)

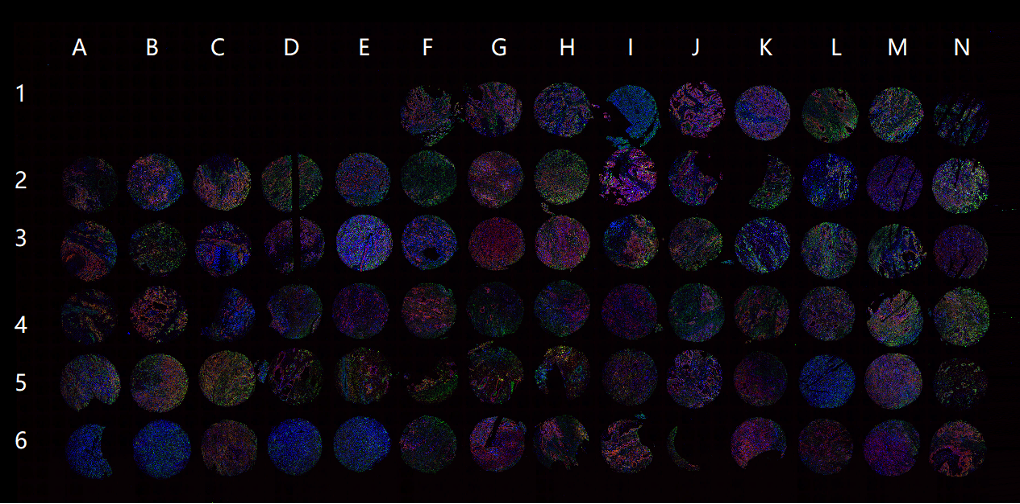

Supplement: Supplementary file 2 [file Image1.jpg]
